# Blockade of the pentraxin 3/CD44 interaction attenuates lung injury‐induced fibrosis

**DOI:** 10.1002/ctm2.1099

**Published:** 2022-11-06

**Authors:** Jhih‐Ying Chi, Yu‐Wei Hsiao, Hsin‐Yin Liang, Tang‐Hsiu Huang, Feng‐Wei Chen, Chen‐Yang Chen, Chiung‐Yuan Ko, Chao‐Chun Cheng, Ju‐Ming Wang

**Affiliations:** ^1^ Department of Biotechnology and Bioindustry Sciences College of Bioscience and Biotechnology National Cheng Kung University Tainan Taiwan; ^2^ Division of Chest Medicine Department of Internal Medicine National Cheng Kung University Hospital College of Medicine National Cheng Kung University Tainan Taiwan; ^3^ Institute of Clinical Medicine College of Medicine National Cheng Kung University Tainan Taiwan; ^4^ Institute of Basic Medical Sciences College of Medicine National Cheng Kung University Tainan Taiwan; ^5^ Ph.D. Program in Medical Neuroscience College of Medical Science and Technology Taipei Medical University Taipei Taiwan; ^6^ International Research Center for Wound Repair and Regeneration National Cheng Kung University Tainan Taiwan; ^7^ Graduate Institute of Medical Sciences College of Medicine Taipei Medical University Taipei Taiwan; ^8^ Graduate Institute of Medicine College of Medicine Kaohsiung Medical University Kaohsiung Taiwan

**Keywords:** CD44, pentraxin 3, pulmonary fibrosis

## Abstract

**Background:**

Fibrosing interstitial lung diseases (fILD) are potentially fatal with limited therapeutic options and no effective strategies to reverse fibrogenesis. Myofibroblasts are chief effector cells in fibrosis that excessively deposit collagen in the pulmonary interstitium and lead to progressive impairment of gaseous exchange.

**Methods:**

Plasma and lung specimens from patients with fILD were applied for detecting pentraxin 3 (PTX3) abundance by ELISA and Immunohistochemistry. Masson's trichrome and Sirius red stains and hydroxyproline assay were performed for assessing collagen accumulation in the lungs of bleomycin‐exposed conditional Ptx3‐deficient and PTX3‐neutralizing antibody (αPTX3i)‐treated mice. Downstream effectors including signaling pathways and fibrotic genes were examined for assessing CD44‐involved PTX3‐induced fibrosis in HFL1 and primary mouse fibroblasts.

**Results:**

PTX3 was upregulated in the lungs and plasma of bleomycin‐exposed mice and correlated with disease severity and adverse outcomes in fILD patients. Decreased collagen accumulation, attenuation of alveolar fibrosis and fibrotic markers, and improved lung function were observed in bleomycin‐exposed conditional Ptx3‐deficient mice. PTX3 activates lung fibroblasts to differentiate towards migrative and highly collagen‐expressing myofibroblasts. Lung fibroblasts with CD44 inactivation attenuated the PI3K‐AKT1, NF‐κB, and JNK signaling pathways and fibrotic markers. αPTX3i mimic‐based therapeutic studies demonstrated abrogation of the migrative fibroblast phenotype and myofibroblast activation in vitro. Notably, αPTX3i inhibited lung fibrosis, reduced collagen deposition, increased mouse survival, and improved lung function in bleomycin‐induced pulmonary fibrosis.

**Conclusions:**

The present study reveals new insights into the involvement of the PTX3/CD44 axis in fibrosis and suggests PTX3 as a promising therapeutic target in fILD patients.

## Graphical abstract

1. PTX3 promotes fibroblast activation and pulmonary fibrosis.
2. CD44 mediates PTX3‐induced fibrotic activation and regulation in lung injury.
3. αPTX3i is a novel and promising candidate for fILD therapy.

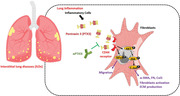

## 1 INTRODUCTION

Interstitial lung diseases (ILDs) refer to a large and heterogeneous group of parenchymal lung disorders. Some ILDs (idiopathic or secondary) tend to manifest as fibrosing ILDs (fILDs) that relentlessly progress towards pulmonary fibrosis despite standard treatments.^1^, ^2^ Idiopathic pulmonary fibrosis (IPF) is the prototype of fILD,^3^ but up to 32% of various other non‐IPF ILDs (particularly idiopathic nonspecific interstitial pneumonia, sarcoidosis, chronic hypersensitivity pneumonitis [cHP], unclassifiable interstitial pneumonia, and certain connective tissue disease [CTD]‐related ILDs) may exhibit the fILD phenotype.^2^ Regardless of the aetiology, distinct ILD subtypes often share overlapping morphological features and common pathological mechanisms. There are currently no effective treatments for fILD and insufficient evidence to guide treatment decisions.^1^ Although antifibrotic agents, such as pirfenidone and nintedanib, have been used as therapies for IPF and certain ILDs with a progressive fibrosing phenotype, these antifibrotic agents did not attenuate or reverse the fibrotic process and may cause side effects.^4^, ^5^ Therefore, exploring effective antifibrotic strategies remains a critical unmet need.

Inflammation is a common link among various pulmonary fibrotic diseases. Persistent inflammatory stimuli and the production of fibrotic cytokines and proinflammatory cytokines during wound healing processes create a microenvironment that further propagates fILD progression, deposits connective‐tissue elements and destroys normal tissue architecture.^6^ Antifibrotic therapies have focused on modulating inflammatory responses and inhibiting myofibroblast activation to decrease extracellular matrix (ECM) deposition.^7^, ^8^ However, current treatments remain far from attaining stronger antifibrotic efficacy in fILD primarily due to the lack of ideal and effective targets to regulate the inflammatory environment and reverse fILD progression.

Pentraxin 3 (PTX3) is a component of the innate immunity humoral arm, and it participates in resistance against inflammation and microorganisms. PTX3 is primarily produced by fibroblasts, phagocytes, neutrophils and endothelial cells during inflammatory processes after the secretion of inflammatory cytokines.^9^ Increasing evidence indicates that PTX3 represents a novel biomarker of clinical inflammation. Elevated PTX3 levels in the blood are observed in several diseases, such as chronic obstructive pulmonary disease,^10^ acute myocardial infarction,^11^ atherosclerotic lesions,^12^ rheumatoid arthritis,^13^ systemic sclerosis^14^ and chronic kidney disease (CKD).^15^ Elevated PTX3 levels are also associated with ECM formation‐induced fibrocyte differentiation in pulmonary fibrosis lesions.^16^ However, the potential for an ideal therapeutic target and details of molecular regulation in tissue remodelling remain poorly understood. The role of PTX3 in lung injury‐associated progressive pulmonary fibrosis has not been elucidated.

CD44 is a transmembrane receptor that is broadly expressed in different tissues and cell types, and it plays major roles in cell–ECM and cell–cell interactions. CD44 interacts with various ligands and growth factor receptors to mediate cellular functions.^17^ CD44 is involved in regulating the inflammatory process. For example, CD44 is upregulated after tissue injury and fibrosis, which activate leukocytes, smooth muscle cells and parenchymal cells at the site of inflammation.^18^ Common phenomena, including a substantially impaired infiltration of macrophages and attenuated accumulation of myofibroblasts, were observed in several disease models using *CD44*‐deficient mice.^19^, ^20^, ^21^ Reconstitution with CD44 significantly attenuated the massive infiltration of inflammatory cells, improved the impaired clearance of apoptotic neutrophils, and ameliorated the continual accumulation of hyaluronan fragments within the alveolar interstitium following lung injury in a *CD44*‐deficient animal model.^22^ These discoveries highlight the importance of characterizing CD44 and identifying CD44‐interacting proteins and their linkages with specific biological functions. We identified CD44 as a direct‐binding receptor of PTX3 on cancer cells.^23^ The involvement of PTX3 in fibrosis regulation has been suggested. However, various issues, including cellular responses to PTX3/CD44 interactions, their participation in pathogenic fibrogenesis in lung diseases and the details of PTX3/CD44‐involved molecular regulation in fibrosis, remain open questions.

## 2 RESULTS

### 2.1 PTX3 is upregulated in fILD patients and bleomycin‐exposed mice

**FIGURE 1:**
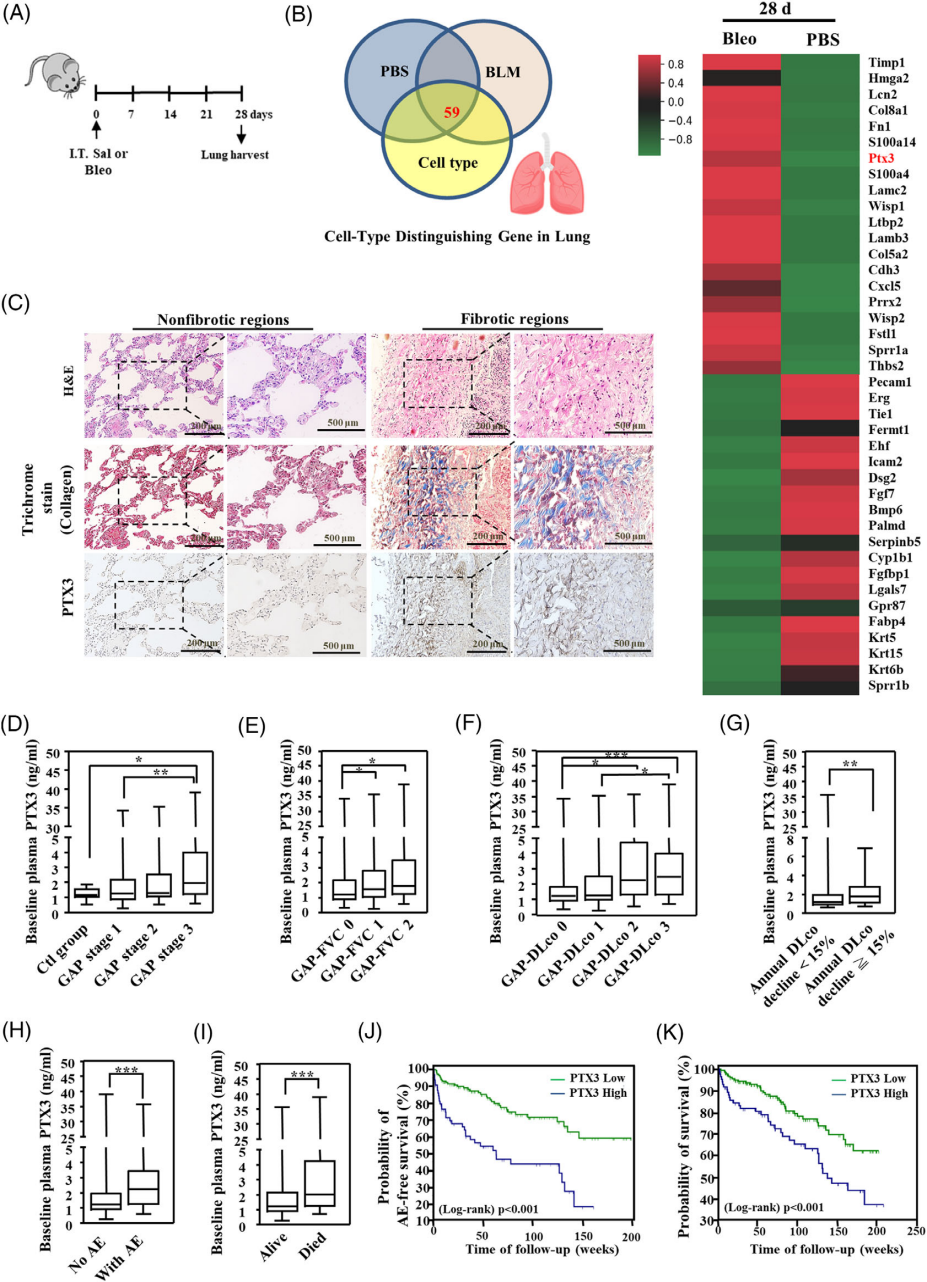
PTX3 is upregulated in the lungs of fILD patients and a murine pulmonary fibrosis model. (A) Scheme showing the experimental setup. C57BL/6 mice were intratracheally instilled with PBS or 2 mg/kg bleomycin (Bleo) on day 0 and subsequently euthanised on days 7, 14, 21, and 28. (B) Venn diagrams representing the number of common up‐ and downregulated genes between sham (phosphate‐buffered saline; PBS) and bleomycin‐exposed mice intersected with the cell‐type dataset. Heatmap representation of the top 40 differentially expressed genes in lungs from PBS‐ and bleomycin‐exposed mice. (C) Representative image of lung sections from pulmonary fibrosis patients with nonfibrotic regions (*n* = 9) and fibrotic lesions (*n* = 30) stained with haematoxylin and eosin (H&E), Masson's trichrome and an anti‐PTX3 antibody. Scale bars are 200 and 500 μm. Comparison of baseline plasma PTX3 levels; (D) between control subjects and patients with fILD of various Gender–Age–Physiology (GAP) stages (control: *n* = 15, GAP1: *n* = 89, GAP2: *n* = 74, GAP3: *n* = 44); (E) between patients with fILD grouped based on scores in the FVC component of the GAP index (GAP‐FVC0: *n* = 86, GAP‐FVC1: *n* = 89, GAP‐FVC2: *n* = 32); (F) between patients with fILD grouped based on scores in the DLco component of the GAP index (GAP‐DLco0: *n* = 110, GAP‐DLco1: *n* = 37, GAP‐DLco2: *n* = 23, GAP‐DLco3: *n* = 37); (G) between patients grouped by the annual DLco exacerbation rate ≥15% or <15% (140 patients had serial DLco measurements, 44 patients had annual rates of decline ≥15%, and 96 patients, <15%); (H) between patients with and without acute exacerbation (AE) during follow‐up (no AE: *n* = 138, with AE: *n* = 69); (I) between patients who died and patients who survived during follow‐up (alive: *n* = 136; died: *n* = 71). Survival curves for patients with baseline plasma PTX3 levels >2.2 (*n* = 65) or <2.2 (*n* = 142) ng/ml with respect to (J) AE‐free survival and (K) overall survival. For panels D to K, differences between the groups were analysed using the Mann–Whitney U test (min–max, median, SD). *p  < .05, **p  < .01, ***p  < .001

Fibrosis is characterised by the deposition of ECM and the destruction of normal tissue architecture. Fibroblasts and epithelial cells are likely major contributors to lung fibrosis.^24^ We established a bleomycin‐induced lung fibrosis animal model (Figure 1A) and performed RNA‐seq analysis to characterise gene changes during pulmonary injury‐associated fibrosis. We combined gene datasets of lung epithelial cells, mesenchymal cells (containing fibroblasts) and endothelial cells in Novartis mouse GeneAtlas to identify potential effectors in lung fibrosis. PTX3 is a regulator in airway mucosal surface homeostasis,^25^ and it is used as a prognostic or disease marker. An increase in the fibrotic markers α‐SMA and fibronectin and PTX3 was observed in lung tissue (Figure 1B and Figure S1A–D), and PTX3 was detectable in the blood plasma (Figure S1E) of bleomycin‐exposed mice. To examine the clinical relationship of PTX3 in pulmonary fibrosis, we further assessed PTX3 expression in patients with fILD and matched control subjects (without fILD). We found that PTX3 was strongly expressed in fibroblastic foci of fibrotic lesions but expressed at low levels in the nonfibrotic region of lung tissues from patients with fILD (Figure 1C). Compared to control subjects, patients with fILD of relatively milder severity (i.e., Gender–Age–Physiology [GAP] stage 1 and stage 2) exhibited comparable baseline plasma PTX3 levels, but patients with the highest severity (GAP 3), exhibited significantly higher baseline plasma PTX3 levels (Figure 1D). Except for older age in fILD patients, no other difference was found in baseline demographic or comorbid characteristics between the two groups (Table S1). However, when all the enrolled subjects were stratified according to age, similar trends of increasing PTX3 with increasing GAP stages were still observed across different age strata (Table S2). The fILD patients with more significant impairment in pulmonary functional parameters (such as forced vital capacity, FVC, and the diffusion capacity of the lung for carbon monoxide, DLco) also exhibited significantly higher baseline plasma PTX3 levels than patients with less impairment in pulmonary function (Figure 1E,F). PTX3 abundance in plasma also correlated with adverse outcomes. Patients with fILD who exhibited an accelerated annual declining rate (>15%) in DLco (Figure 1G), patients who developed acute exacerbation (AE) during follow‐up (Figure 1H), and patients who died during follow‐up (Figure 1I) had significantly higher baseline plasma PTX3 levels than patients who did not experience these adverse outcomes. Using 2.2 ng/ml as a cut‐off value (which was derived from receiver operating characteristic [ROC] analyses), patients with fILD and baseline plasma PTX3 levels ≥2.2 ng/ml had significantly lower probabilities of AE‐free survival (Figure 1J) and overall survival (Figure 1K) and significantly increased crude and adjusted hazard ratios for AEs and all‐cause mortality (Table 1) than patients with baseline plasma PTX3 levels <2.2 ng/ml.

**TABLE 1:** Crude and adjusted hazard ratios for AE and mortality when having a baseline plasma PTX3 level > 2.2 ng/ml

Cox proportional hazard regression showed that patients having baseline PTX3 > 2.2 ng/ml had significantly higher risk of developing acute exacerbation and death during follow‐up than patients with baseline PTX3 < 2.2 ng/ml.	
**Acute exacerbation**	
**Crude HR (95% CI)**	3.12 (1.94–5.01) *p* < .001
**Adjusted HR (95% CI)** **^#^**	2.55 (1.51–4.31) *p* < .001
**Death**	
**Crude HR (95% CI)**	2.18 (1.35–3.52) *p* = .001
**Adjusted HR (95% CI)** **^#^**	1.70 (1.01–2.86) *p* = .047

Abbreviations: CI, confidence interval; HR, hazard ratio.

^#^ In multi‐variable Cox proportional hazard regression, adjustment was made for the following co‐variables: age, sex, smoking status, echocardiographic pulmonary hypertension, and Charlson comorbidity index.

### 2.2 Conditional knockout of *Ptx3* reverses bleomycin‐induced pulmonary fibrosis

**FIGURE 2:**
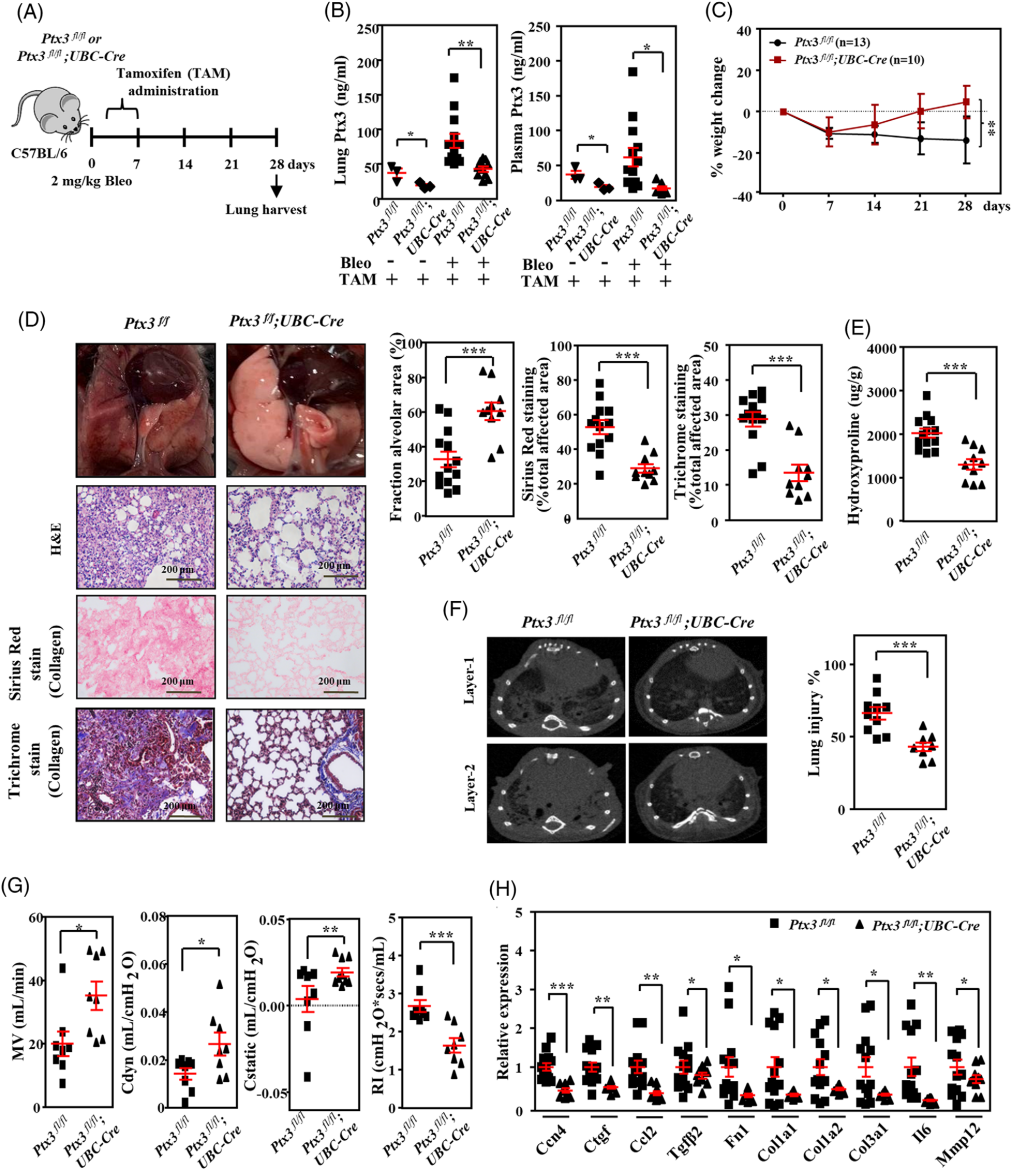
Bleomycin‐induced pulmonary fibrosis is attenuated in conditional *Ptx3* knockout mice. (A) Scheme showing the experimental setup in tamoxifen‐induced conditional *Ptx3*‐knockout mice. Wild‐type *(Ptx3^fl/fl^)* mice (*n* = 13) and tamoxifen‐induced conditional *Ptx3*‐knockout *(Ptx3^fl/fl^;UBC‐Cre)* mice (*n* = 10) were intratracheally instilled with 2 mg/kg bleomycin (Bleo) on day 0 and subsequently euthanised on day 28. Tamoxifen was administered to *Ptx3^fl/fl^* and *Ptx3^fl/fl^; UBC‐Cre* mice daily from days 3 to 7 via intraperitoneal injection. (B) Lung tissue Ptx3 and plasma Ptx3 concentrations were measured in *Ptx3^fl/fl^* mice and *Ptx3^fl/fl^;UBC‐Cre* mice on day 28 with or without bleomycin administration using ELISA. (C) Body weight was estimated for 28 days post‐bleomycin administration as a percentage of day 0 weight for each mouse group as indicated. (D) Macroscopic view of the lungs at the study endpoint. Representative haematoxylin and eosin (H&E), Sirius red and Masson's trichrome staining in representative lung sections from *Ptx3^fl/fl^* mice (*n* = 13) and *Ptx3^fl/fl^;UBC‐Cre* mice (*n* = 10) on day 28 post‐bleomycin administration. Scale bars are 200 μm. Quantitative analysis of the alveolar area was performed on H&E‐stained sections, and the fibrosis area was quantified in Sirius red‐ and Masson's trichrome‐stained sections of lung tissue. (E) Lung hydroxyproline concentrations were measured using a hydroxyproline assay kit. Lung tissue was harvested from *Ptx3^fl/fl^* mice (*n* = 13) and *Ptx3^fl/fl^;UBC‐Cre* mice (*n* = 10) after bleomycin administration. (F) Representative computed tomography slices of mouse lungs from *Ptx3^fl/fl^* mice (*n* = 10) and *Ptx3^fl/fl^;UBC‐Cre* mice (*n* = 8) on day 28 post‐bleomycin administration. Quantitative analysis of lung injury was performed on micro‐CT sections using CT‐Analyzer software. (G) Total respiratory system lung resistance (Rl), dynamic compliance (Cdyn), static lung compliance (Cstatic), lung elastance (E) and minute volume (MV) were measured from *Ptx3^fl/fl^* mice (*n* = 18) and *Ptx3^fl/fl^;UBC‐Cre* mice (*n* = 8) on day 28 post‐bleomycin administration. (H) Representative fibrosis‐related genes *Fn1, Col1a1, Col1a2, Col3a1, Ctgf, Mmp12, Il6, Tgfb2, Ccl2* and *Ccn4* were quantified in lung homogenates isolated from *Ptx3^fl/fl^* mice (*n* = 13) and *Ptx3^fl/fl^;UBC‐Cre* mice (*n* = 10) on day 28 post‐bleomycin administration using qRT–PCR. All data are shown as the means  ±  SEM. Differences between groups were analysed using unpaired two‐tailed *t*‐tests or one‐way ANOVA followed by Tukey's multiple comparison test. *p  < .05, **p  < .01, ***p  < .001

To determine the contributions of PTX3 to pathological fibrogenesis in vivo, tamoxifen‐induced *Ptx3*‐deficient mice were used in a bleomycin‐induced pulmonary fibrosis model (Figure 2A). Following confirmation of the attenuation of PTX3 in *Ptx3*‐deficient mice (Figure 2B), significant body weight recovery was observed in bleomycin‐exposed *Ptx3*‐deficient mice compared to *Ptx3* wild‐type mice (Figure 2C). Compared to *Ptx3* wild‐type mice, end‐point haematoxylin and eosin (H&E) staining of alveolar morphology revealed reduced lung injury in bleomycin‐exposed *Ptx3‐*deficient mice (Figure 2D). Compared to the lungs of *Ptx3* wild‐type mice, Sirius red and Masson's trichome staining revealed a consistent result: significantly attenuated bleomycin‐induced collagen deposition in bleomycin‐exposed *Ptx3‐*deficient mice (Figure 2D). Compared to *Ptx3* wild‐type mice, the hydroxyproline level was significantly reduced in bleomycin‐exposed *Ptx3‐*deficient mice (Figure 2E). Consistent with these histological observations, *Ptx3‐*deficient mice exhibited a reduced volume of fibrotic lesions as assessed by microcomputed tomography (micro‐CT) images (Figure 2F) and significantly improved pulmonary function in minute volume (MV), dynamic compliance (Cdyn), static lung compliance (Cstatic) and lung resistance (Rl) in body plethysmography (Figure 2G). The expression of fibrogenesis‐related genes was attenuated in the lung tissues of bleomycin‐exposed *Ptx3‐*deficient mice (Figure 2H). These results suggest that the inactivation of PTX3 attenuates the progression of pulmonary fibrosis in vivo. Moreover, inducible fibroblast‐specific *Ptx3*‐deficient (*Ptx3^fl/fl^;Col1a2‐Cre‐ERT2*) mice were generated and used to assess the contribution of fibroblast PTX3 to bleomycin‐induced lung injury and fibrosis (Figure S2A–C). Following the administration of tamoxifen, although attenuated body weight loss was observed (Figure S2D,E), no significant change of plasma Ptx3 (Figure S2F) and no improvement in the volume of fibrotic lesions (Figure S2G) or pulmonary function (Figure S2H) was observed in bleomycin‐induced *Ptx3^fl/fl^;Col1a2‐Cre‐ERT2* mice. This implies that PTX3 from fibroblast may not be sufficient to enhance bleomycin‐induced lung injury.

### 2.3 PTX3/CD44 signalling contributes to myofibroblast differentiation and lung fibrosis

**FIGURE 3:**
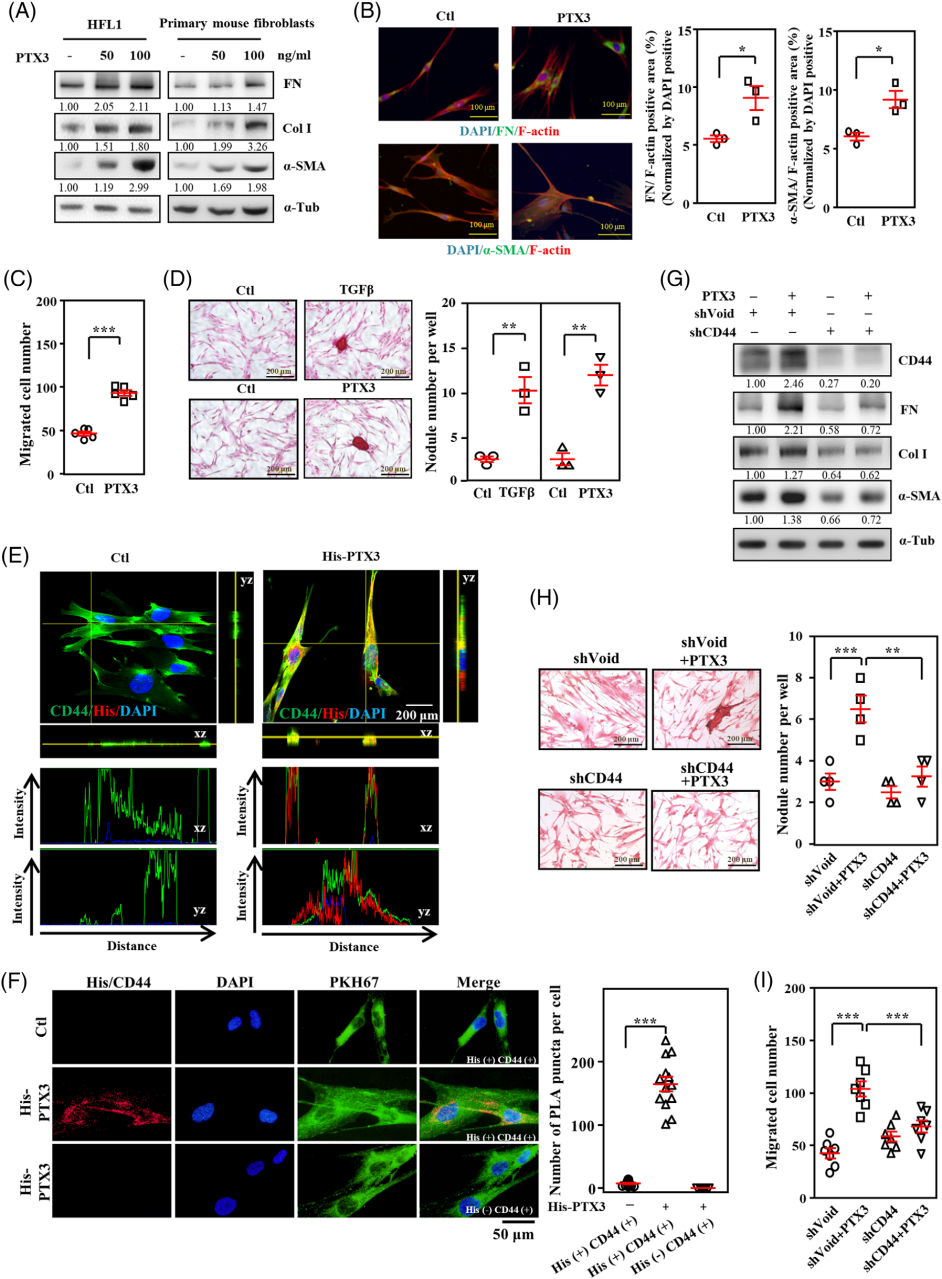
PTX3 signalling activates myofibroblast differentiation and lung fibrosis. (A) Primary mouse lung fibroblasts or human lung fibroblasts (HFL1) were treated with or without 50 ng/ml or 100 ng/ml PTX3 for 6 h. Lysates from the experimental cells were harvested and analysed using immunoblotting with specific antibodies as indicated. α‐Tubulin was used for relative protein expression normalization. Immunoblotting was replicated independently at least three times per experiment. (B) HFL1 cells were treated with or without 100 ng/ml PTX3 for 6 h and subjected to immunostaining for fibronectin, α‐SMA and F‐actin. Scale bars are 100 μm. Quantification of the fibronectin/F‐actin‐positive area or α‐SMA/F‐actin‐positive area normalised to the DAPI‐positive area. (C) The migratory capacity of fibroblasts was evaluated by determining the number of HFL1 cells treated with or without 100 ng/ml PTX3 for 18 h. (D) The nodule collagen formation of fibroblasts was determined by counting the nodule number in HFL1 cells treated with or without 100 ng/ml PTX3 or 5 ng/ml TGFβ for 24 h. Scale bars are 200 μm. (E) After staining with the indicated antibodies, PTX3‐treated HFL1 cells were immobilised and detected using immunofluorescence and confocal microscopy. Representative immunostaining of HFL1 cells with merged 2D images. (F) The interaction between His‐PTX3 and CD44 was determined by in situ PLA using anti‐His and anti‐CD44 antibodies (His+, CD44+). The red spots represent interacting complexes of His‐PTX3 and CD44. Cells stained with anti‐CD44 antibody only (His‐, CD44+) were used as a negative control. The nuclei were stained with DAPI (blue). The cell membrane was stained with PKH67. Protein interactions were quantified by counting the number of puncta per cell. (G) HFL1 cells were infected with control (shVoid) or shCD44 lentiviruses and treated with PTX3 for 6 h. Lysates from experimental cells were harvested for Western blot analysis, and specific antibodies were applied as indicated. Relative protein expression was normalised to α‐tubulin. Immunoblotting was replicated independently at least three times per experiment. (H) The nodule collagen formation of fibroblasts was determined by counting the nodule number in HFL1 cells infected with shVoid or shCD44 lentiviruses and treated with or without PTX3 for 24 h. (I) The migrative capacity of fibroblasts was estimated by counting the number of HFL1 cells infected with shVoid and shCD44 lentiviruses treated with or without PTX3 for 18 h. All data are expressed as the means  ±  SEM. Differences between the groups were analysed using unpaired two‐tailed *t*‐tests or one‐way ANOVA followed by Tukey's multiple comparison test. *p  < .05, **p  < .01, ***p  < .001

Myofibroblasts are critical effector cells of excessive ECM deposition in pulmonary fibrosis.^26^ We examined whether PTX3 participated in the activation of myofibroblast differentiation and activation. Treatment with recombinant PTX3 protein^25^ activated fibronectin, collagen I and α‐SMA stress fibre organization in human lung fibroblast cells (HFL1) and primary mouse lung fibroblasts (Figure 3A,B and Figure S3A). We examined whether PTX3 contributed to fibroblast proliferation, migration and in vitro nodule formation.^27^ The results showed that PTX3 had no effect on the proliferation of HFL1 cells (Figure S3B) but enhanced fibroblast migration (Figure 3C), activation and in vitro fibrotic nodule formation (Figure 3D). CD44 regulates inflammatory responses and lung fibrosis.^22^, ^28^, ^29^ We previously identified CD44 as a PTX3 direct‐binding receptor.^25^ However, whether the PTX3/CD44 complex plays a vital role in pathogenic fibrogenesis in the lung and the details of PTX3/CD44 molecular regulation in tissue remodelling are not known. The direct binding between PTX3 and CD44 was examined using several in vitro binding assays. The ELISA results showed that recombinant PTX3 proteins interacted with recombinant CD44 proteins (Figure S4A). The immunofluorescent PTX3 signals colocalised with the immunofluorescent CD44 signals on HFL1 cell membranes (Figure 3E). A proximity ligation assay (PLA) was performed to verify the interaction and association between PTX3 and activated CD44 using PTX3 and CD44 antibodies. Compared to without recombinant PTX3 protein treatment, an increased fluorescent signal in response to the interaction of recombinant PTX3 protein and active CD44 on the cell membrane was observed on the HFL1 cell membrane (Figure 3F). The results of PTX3/CD44 interaction assays suggested that PTX3 interacted with CD44 on HFL1 cells. We next assessed whether CD44 mediated PTX3‐induced profibrogenic effects. PTX3 treatment‐induced increases in fibrotic markers, including fibronectin, collagen I and α‐SMA (Figure 3G and Figure S4B–D), and fibrotic characteristics, including in vitro fibrotic nodule formation (Figure 3H) and migration (Figure 3I), were attenuated in CD44 knockdown cells, which indicates that CD44 mediates PTX3‐induced profibrotic activity.

### 2.4 Elucidation of PTX3/CD44‐induced signalling pathways in fibroblast activation

**FIGURE 4:**
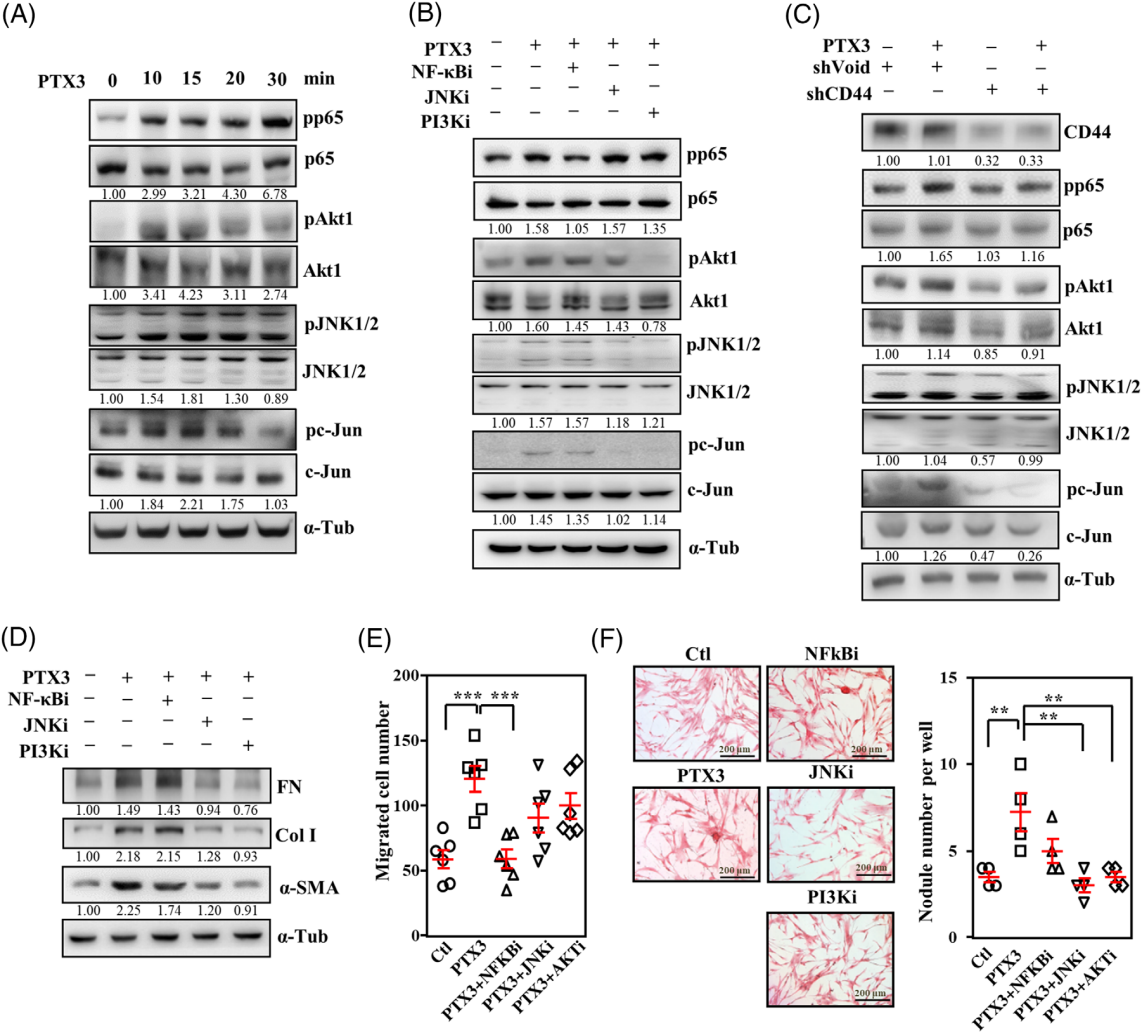
Disruption of the PTX3 and CD44 interaction attenuates fibrosis signalling. (A) The activity of AKT1, JNK, c‐Jun and NF‐κB (p65) in response to PTX3 treatment over the indicated time courses. Lysates from experimental cells were harvested for Western blot analysis. (B) HFL1 cells were pre‐treated with or without wortmannin (50 nM), BAY 11–7085 (1 μM) or JNK inhibitor II (7.5 μM) for 10 min and treated with or without PTX3 for 15 min. Cell lysates were immunoblotted for pAKT1, pJNK, pc‐Jun and pp65 and normalised to the respective protein levels. α‐Tubulin served as the internal control. (C) HFL1 cells were infected with shVoid or shCD44 lentiviruses and treated with PTX3 for 15 min. The activity of AKT1, JNK, c‐Jun and p65 was examined using Western blotting and normalised to the respective protein levels. α‐Tubulin was used as the internal control. (D) The indicated signalling inhibitors were pre‐treated before treatment with or without PTX3 for 24 h. Lysates from experimental cells were harvested for Western blot analysis using specific antibodies as indicated. Relative protein expression was normalised to α‐tubulin. Immunoblotting was replicated independently at least three times per experiment. (E) The migratory capacity of fibroblasts was assessed by determining the number of HFL1 cells following pre‐treatment with the indicated signalling inhibitors and then treatment with or without PTX3 for 18 h. (F) The nodule collagen formation in fibroblasts was determined by counting the nodule number in HFL1 cells following pre‐treatment with the indicated signalling inhibitors and then treatment with or without PTX3 for 24 h. Scale bars are 100 μm. All data are shown as the means  ±  SEM. Differences between groups were analysed using one‐way ANOVA followed by Tukey's multiple comparison test. **p  < .01, ***p  < .001

PI3K/AKT1, MAPKs, and NF‐κB signalling are activated in response to inflammatory factors and contribute to fibrotic processes, such as proliferation, differentiation, ECM synthesis and migration.^30^, ^31^, ^32^ PTX3 treatment activated NF‐κB, AKT1, JNK1/2, and c‐Jun in HFL1 cells and primary mouse lung fibroblasts (Figure 4A and Figure S5A–C). Three specific pharmacological inhibitors, including PI3K/AKT1 (wortmannin), NF‐κB (BAY 11–7085) and JNK1/2 inhibitor (SP600125), were used to elucidate the hierarchy and involvement of NF‐κB, AKT1, and JNK1/2 signalling upon PTX3 treatment. Briefly, the results showed that BAY 11–7085 specifically inhibited NF‐κB p65 activation but not JNK1/2/c‐Jun or AKT1 activation. SP600125 specifically inhibited JNK/c‐Jun activation but not AKT1 activation; and wortmannin specifically inhibited AKT1 and JNK/c‐Jun activation but not NF‐κB p65 activation (Figure 4B and Figure S5D–F). These results suggested that PTX3 induced at least two independent pathways, NF‐κB and PI3K/AKT1/JNK1/2/c‐Jun, in lung fibroblasts. The loss of CD44 attenuated these two independent pathways (Figure 4C and Figure S6A–C). Wortmannin and SP600125, but not BAY 11–7085, inhibited PTX3‐induced fibronectin, collagen I, and α‐SMA (Figure 4D and Figure S6D–F). However, BAY 11–7085 but not wortmannin or SP600125 specifically inhibited the migration of fibroblasts (Figure 4E), and wortmannin and SP600125 but not BAY 11–7085 attenuated fibrotic nodule formation (Figure 4F). These results suggest that the NF‐κB and PI3K/AKT1/JNK1/2/c‐Jun pathways affect different fibrotic features in response to the PTX3/CD44 interaction in HFL1 cells.

### 2.5 Blockade of PTX3 inhibits bleomycin‐induced pulmonary fibrosis in vivo

**FIGURE 5:**
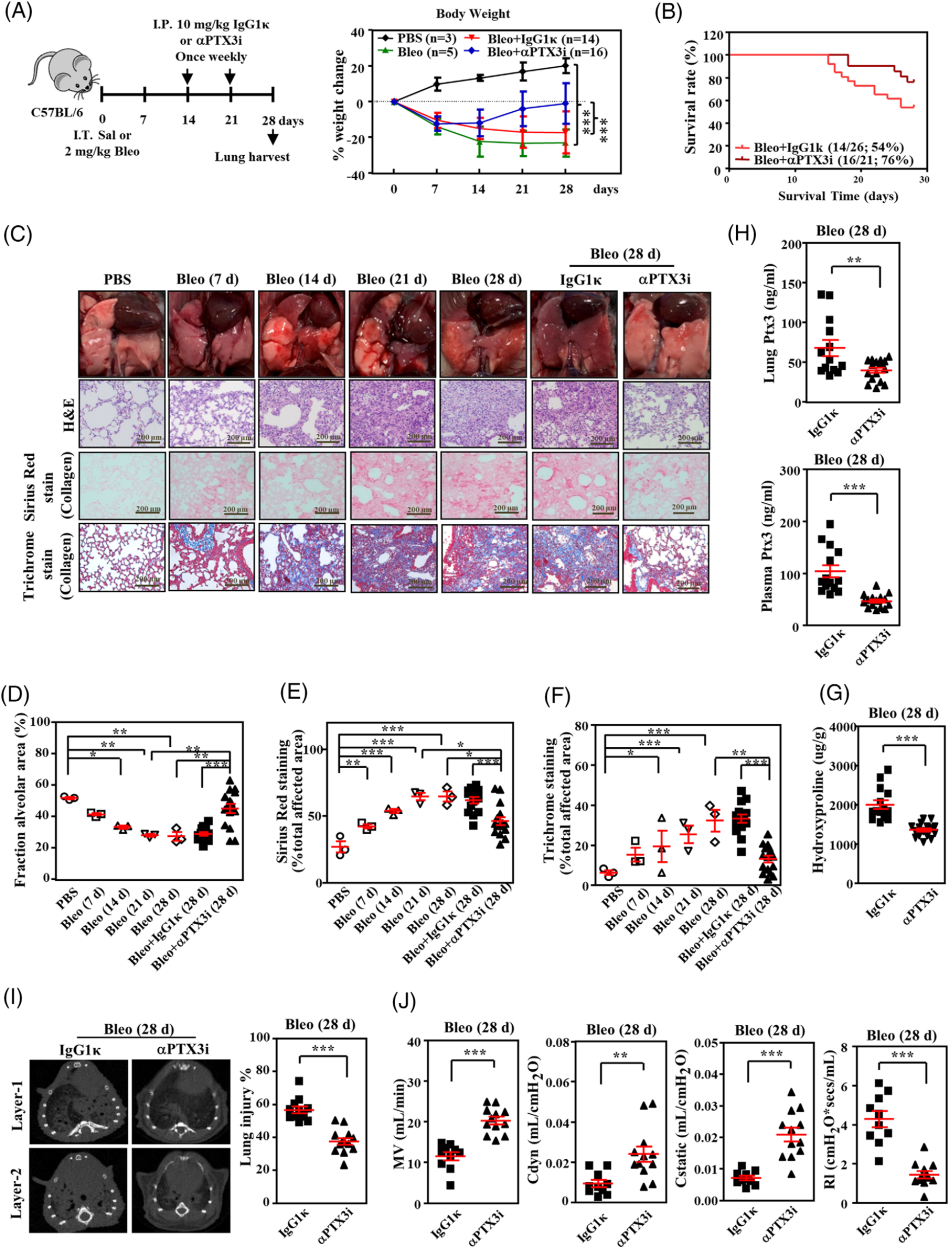
αPTX3i significantly attenuates bleomycin‐induced pulmonary fibrosis in mice. (A) Scheme showing the experimental setup. C57BL/6 mice were intratracheally instilled with PBS or 2 mg/kg bleomycin (Bleo) on day 0 and euthanised on day 28. IgG1κ (10 mg/kg) or αPTX3i (10 mg/kg) was administered on days 14 and 21 via intraperitoneal injection. Body weight was estimated for 28 days post‐bleomycin administration as a percentage of day 0 weight for each mouse group as indicated. (PBS: *n* = 3, Bleo: *n* = 5, Bleo + IgG1κ: *n* = 14, Bleo + αPTX3i: *n* = 16). (B) Percentages of surviving mice plotted over the 28‐day period: IgG1κ group and αPTX3i group. (C) C57BL/6 mice were intratracheally instilled with PBS or 2 mg/kg bleomycin (Bleo) on day 0 and euthanised on days 7, 14, 21, and 28 (*n* = 3, each group). Macroscopic view of the lungs at the study endpoint. Representative haematoxylin and eosin (H&E), Sirius red and Masson's trichrome staining in representative lung sections from IgG1κ‐treated mice (*n* = 14) and αPTX3i‐treated mice (*n* = 16) post‐bleomycin administration. Scale bars are 200 μm. (D) Quantitative analysis of the alveolar area was performed on H&E‐stained sections. (E,F) The area of fibrosis was quantified using Sirius red‐ and Masson's trichrome‐stained sections of lung tissue. (G) Lung hydroxyproline concentrations were measured using a hydroxyproline assay kit. Lung tissue was harvested from IgG1κ‐treated mice (*n* = 14) and αPTX3i‐treated mice (*n* = 16) after bleomycin administration. (H) Lung tissue Ptx3 and plasma Ptx3 concentrations were measured using ELISA. Lung tissue and plasma were harvested from IgG1κ‐treated mice (*n* = 14) and αPTX3i‐treated mice (*n* = 16) after bleomycin administration. (I) Representative computed tomography slices of mouse lungs on day 28 from IgG1κ‐treated mice (*n* = 12) and αPTX3i‐treated mice (*n* = 13) post‐bleomycin administration. Quantitative analysis of lung injury was performed on micro‐CT sections using CT‐Analyzer software. (J) The function of the respiratory system, including minute volume (MV), dynamic compliance (Cdyn), static lung compliance (Cstatic) and lung resistance (Rl), was measured on day 28 in IgG1κ‐treated (*n* = 10) and αPTX3i‐treated mice (*n* = 12) after bleomycin administration. All data are shown as the means  ±  SEM. Differences between groups were analysed using unpaired two‐tailed *t*‐tests or one‐way ANOVA followed by Tukey's multiple comparison test. *p  < .05, **p  < .01, ***p  < .001

Our results suggested that disruption of the PTX3 and CD44 interaction attenuated lung fibroblast activation. Therefore, a neutralised monoclonal anti‐PTX3 antibody (αPTX3i)^25^ that interrupts PTX3 and CD44 interactions was used to investigate whether the inhibition of PTX3/CD44 attenuated lung injury‐induced fibrosis. Assessment of the toxicity and safety of αPTX3i in vivo and in vitro assays revealed no adverse effects or effects on cell survival (Figure S7A–G). Therefore, the effects of αPTX3i on bleomycin‐induced pulmonary fibrosis were investigated. Dose‐dependent recovery of body weight and prolonged overall animal survival following treatment with various doses of αPTX3i in bleomycin‐induced mice were observed (Figure 5A,B and Figure S8A,B). Consistent with the attenuated phenomena observed in bleomycin‐exposed inducible *Ptx3*‐deficient mice, the severity and quantification parameters of lung histology, collagen fibre accumulation and hydroxyproline content were significantly attenuated following αPTX3i treatment (Figure 5C–G and Figure S9). Notably, milder pulmonary fibrosis was observed in the αPTX3i treatment groups on day 28 than on day 14 after αPTX3i injection, which indicated that αPTX3i potentially reversed fibrotic progression. After bleomycin exposure, lung PTX3 and plasma PTX3 levels were strikingly lower in the αPTX3i group than the IgG1κ group (Figure 5H). This result implies that PTX3 is autoregulated in lung injury‐induced fibrosis. Ex vivo micro‐CT and pulmonary function assays were performed to assess the effects of αPTX3i. The results showed that αPTX3i significantly reduced damage and lung densities on micro‐CT (Figure 5I and Figure S8C) and recovered pulmonary function (Figure 5J and Figure S8D) in bleomycin‐exposed mice.

### 2.6 Blockade of PTX3 inhibits lung fibroblast activation in vitro

**FIGURE 6:**
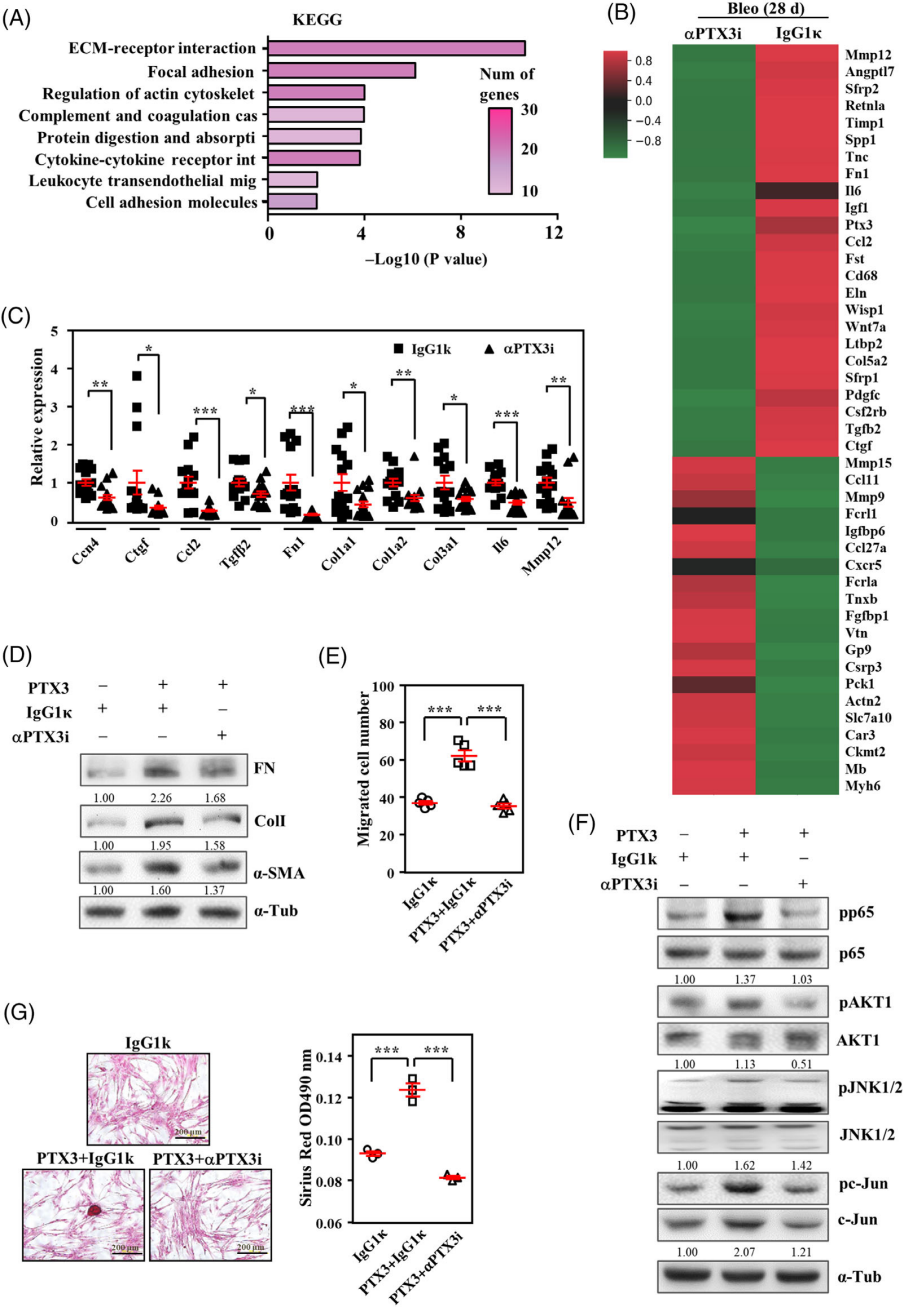
αPTX3i inhibits PTX3‐induced regulation of lung fibroblasts. (A) KEGG‐based enrichment analysis of altered transcripts in bleomycin‐exposed mice versus PBS‐treated mice compared to IgG1κ‐treated mice versus αPTX3i‐treated mice using the DAVID bioinformatics tool and plotted by highest significance (−log10 of modified Fisher's exact *p* value). The colour of the bar represents the number of genes in each group according to the legend. (B) Heatmap representation of the top 50 differentially expressed genes in lungs from IgG1κ‐treated and αPTX3i‐treated mice following bleomycin administration. (C) Representative fibrosis‐related genes were quantified using qRT–PCR of total RNA extracted from lung homogenates isolated from IgG1κ‐treated (*n* = 14) and αPTX3i‐treated mice (*n* = 16) post‐bleomycin administration. (D) HFL1 cells were preincubated with IgG1κ and αPTX3i and then treated with PTX3 for 6 h. Lysates from experimental cells were harvested for Western blot analysis using the indicated antibodies. α‐Tubulin was used for relative protein expression normalization. Immunoblotting was replicated independently at least three times per experiment. (E) The migratory capacity of fibroblasts was assessed by determining the number of HFL1 cells preincubated with IgG1κ and αPTX3i followed by treatment with PTX3 for 18 h. (F) HFL1 cells were incubated with IgG1κ and αPTX3i and then treated with PTX3 for 15 min. The activity of AKT1, JNK, c‐Jun and NF‐κB (p65) was assessed using Western blotting, and the intensity of images was normalised to their respective protein. α‐Tubulin was used as an internal control. Immunoblotting was replicated independently at least three times per experiment. (G) The nodule collagen formation of fibroblasts was determined by counting the nodule number in HFL1 cells preincubated with IgG1κ and αPTX3i and then treated with PTX3 for 24 h. Scale bars are 200 μm. All data are shown as the means  ±  SEM. Differences between the groups were analysed using unpaired two‐tailed *t*‐tests or one‐way ANOVA followed by Tukey's multiple comparison test. *p  < .05, **p  < .01, ***p  < .001

To further investigate the effectors that respond to αPTX3i treatment in bleomycin‐induced pulmonary fibrosis in vivo and verify the responses in vitro, RNA‐seq gene profiling was performed using tissue samples after treatment with αPTX3i or IgG1κ. We performed KEGG pathway enrichment analysis to characterise the signalling pathways responding to αPTX3i administration in bleomycin‐exposed mice. Notably, the results were consistent with our above observations and suggested that PTX3 contributed to adhesion‐, fibrosis‐, migration‐ and immune‐associated signalling pathways (Figure 6A). Several genes were assessed using the lung tissues of experimental mice (Figure 6B,C). In vitro assays assessed the effects of αPTX3i in HFL1 cells. Consistent with the above results on the inactivation of CD44 in lung fibroblasts, αPTX3i inhibited PTX3/CD44‐induced fibrotic marker genes, signalling pathways, and fibrotic features, including fibroblast migration and nodule formation (Figure 6D–G and Figure S10A–D).

### 2.7 αPTX3i is a potential candidate drug for the treatment of lung injury‐induced fibrosis

**FIGURE 7:**
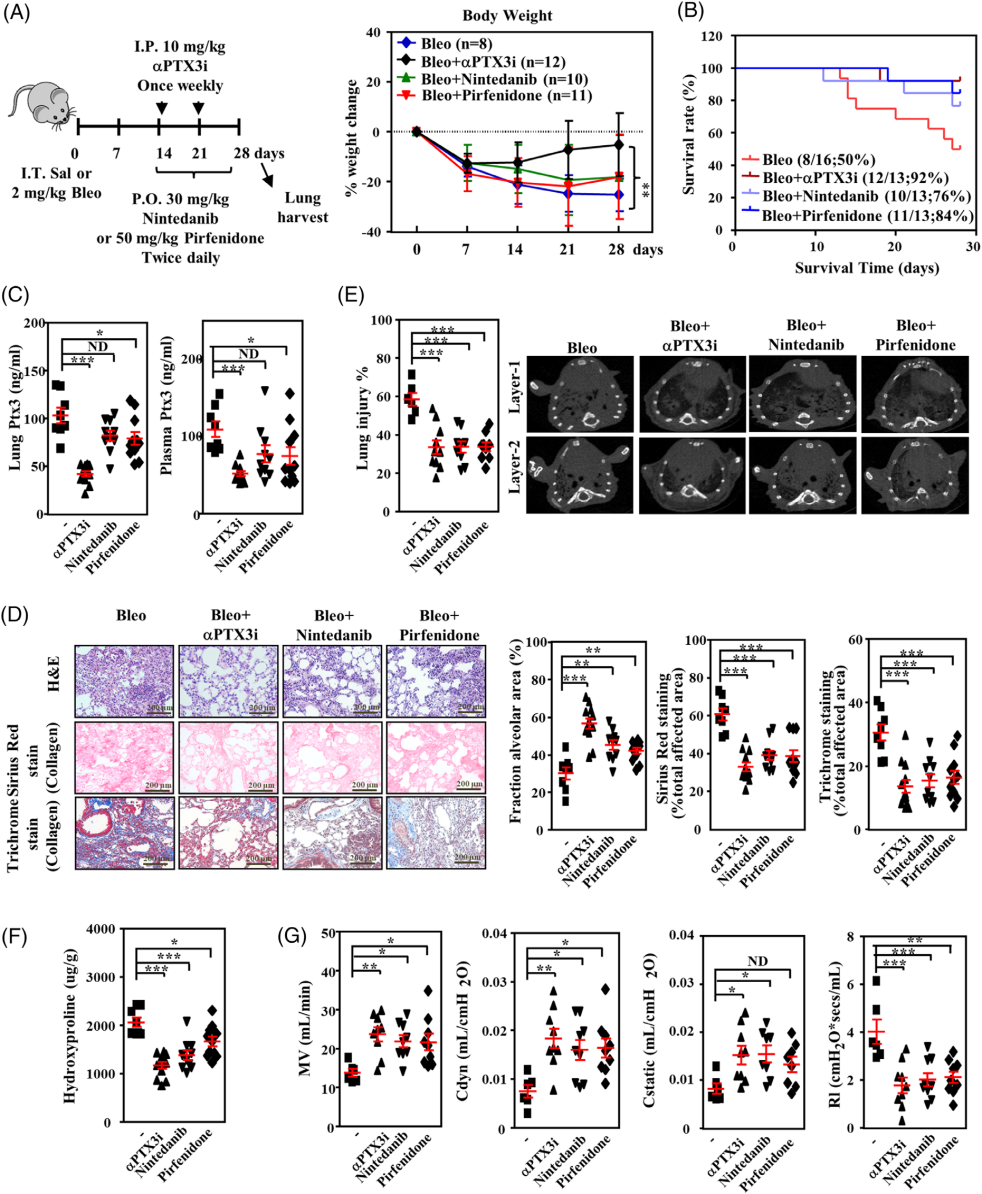
The effect of pirfenidone, nintedanib and αPTX3i in bleomycin‐induced pulmonary fibrosis. (A) Scheme showing the experimental flowchart. C57BL/6 mice were intratracheally instilled with 2 mg/kg bleomycin (Bleo) on day 0 and euthanised on day 28. αPTX3i (10 mg/kg) was administered on days 14 and 21 via intraperitoneal injection. Nintedanib (30 mg/kg) or pirfenidone (50 mg/kg) was orally administered twice daily beginning on day 14 and continued until dissection. Body weight was estimated for 28 days post‐bleomycin administration as a percentage of day 0 weight for each mouse group as indicated. (Bleo: *n* = 8, Bleo + αPTX3i: *n* = 12, Bleo + nintedanib: *n* = 10, Bleo + pirfenidone: *n* = 11). (B) Percentages of surviving mice plotted over a 28‐d period: bleomycin group, αPTX3i group, Nintedanib group and Pirfenidone group. (C) Lung tissue Ptx3 and plasma Ptx3 concentrations were measured using ELISA. Lung tissue and plasma were harvested from vehicle‐ (*n* = 8), αPTX3i‐ (*n* = 12), nintedanib‐ (*n* = 10) and pirfenidone‐treated mice (*n* = 11) following bleomycin administration. (D) Representative haematoxylin and eosin (H&E), Sirius red and Masson's trichrome staining in representative lung sections from vehicle‐ (*n* = 8), αPTX3i‐ (*n* = 12), nintedanib‐ (*n* = 10) and pirfenidone‐treated mice (*n* = 11) following bleomycin administration. Scale bars are 200 μm. Quantitative analysis of the alveolar area was performed on H&E‐stained sections. The area of fibrosis was quantified using Sirius red‐ and Masson's trichrome‐stained sections of lung tissues. (E) Representative computed tomography slices of mouse lungs on day 28 from the vehicle (*n* = 6), αPTX3i (*n* = 10), nintedanib (*n* = 9) and pirfenidone groups (*n* = 9) following bleomycin administration. Quantitative analysis of lung injury was performed on micro‐CT sections using CT‐Analyzer software. (F) Lung hydroxyproline concentrations were measured using a hydroxyproline assay kit. Lung tissue was harvested from the vehicle (*n* = 8), αPTX3i (*n* = 12), nintedanib (*n* = 10) and pirfenidone groups (*n* = 11) following bleomycin administration. (G) The function of the total respiratory system, including minute volume (MV), dynamic compliance (Cdyn), static lung compliance (Cstatic) and lung resistance (Rl), was measured on day 28 in the vehicle (*n* = 6), αPTX3i (*n* = 9), nintedanib (*n* = 9) and pirfenidone groups (*n* = 9) following bleomycin administration. All data are shown as the means  ±  SEM. Differences between the groups were analysed using one‐way ANOVA followed by Tukey's multiple comparison test. *p  < .05, **p  < .01, ***p  < .001

Pirfenidone and nintedanib are FDA‐approved first‐line antifibrotic treatments for IPF and certain fILDs. However, these drugs at best slow, but do not reverse disease progression. These drugs also cause disturbing side effects.^1^ Therefore, we examined the efficacy of αPTX3i compared to pirfenidone and nintedanib in lung injury‐induced fibrosis. The administration of αPTX3i (10 mg/kg/week) significantly recovered body weight loss (Figure 7A), prolonged overall animal survival (Figure 7B), reduced lung and plasma Ptx3 (Figure 7C), reduced the severity of pulmonary fibrosis as examined by H&E staining, Sirius red staining and Masson's staining (Figure 7D), decreased the extent of pulmonary damage (Figure 7E) and content of hydroxyproline (Figure 7F) and improved pulmonary function (Figure 7G) compared to pirfenidone (100 mg/kg/day) and nintedanib (60 mg/kg/day) in bleomycin‐exposed mice.

**FIGURE 8:**
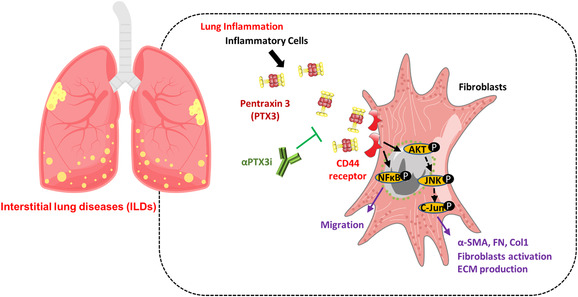
Model for the role of PTX3 in fibrosing interstitial lung diseases (fILDs) by CD44‐regulated fibrotic signalling PTX3 is upregulated in the lungs of bleomycin‐treated mice and fILD patients. PTX3 activates lung fibroblasts to differentiate towards migrative and highly collagen‐expressing myofibroblasts. Moreover, lung fibroblasts with CD44 deficiency exhibited no response to PTX3‐activated PI3K‐AKT, NF‐κB and JNK signalling pathways and diminished fibrotic markers. In therapeutic studies, PTX3‐neutralizing antibody (αPTX3i) disrupted the interaction between PTX3 and CD44, further abrogating the migrative fibroblast phenotype and impeding myofibroblast activation in vitro. Remarkably, αPTX3i diminished lung fibrosis, reduced collagen deposition, increased mouse survival and improved lung function in bleomycin‐induced pulmonary fibrosis

In summary, we revealed that PTX3 is upregulated in the lungs of bleomycin‐exposed mice and fILD patients. Lung injury and fibrosis were significantly attenuated in inducible conditional *Ptx3*‐deficient mice in response to bleomycin exposure. Moreover, we dissected mechanistic insights into PTX3/CD44‐dependent fibrotic pathways and effectors in lung myofibroblast activation and collagen production. Importantly, αPTX3i disrupts the interaction of PTX3 and CD44 and effectively attenuated bleomycin‐treated lung injury and fibrosis in vivo and myofibroblast activation in vitro. Taken together, our study provides new insight into the regulation of PTX3 in pulmonary fibrosis and a potential target for developing a future novel therapy for fILDs (Figure 8).

## 3 DISCUSSION

TGFβ is the most well‐characterised profibrotic factor, and it will likely become a target in the treatment of fibrosis. However, TGFβ has a wide range of biological functions, and inhibition of TGFβ may lead to adverse reactions and affect treatment efficacy. Therefore, in addition to optimizing TGFβ‐associated antifibrotic strategies or drugs, there is a large need to identify and reveal novel profibrotic factors for the development of more efficient and safe therapeutics for fibrotic diseases. WNT signalling pathways are also key regulators of fibrosis.^33^, ^34^ Connective tissue growth factor (CTGF) is an essential mediator of the fibrotic activity of TGFβ and WNT.^35^, ^36^ PTX3 induces TGFβ expression in macrophages.^37^ Our microarray profiling revealed that CTGF, TGFβ and WNT were responsive to bleomycin and were attenuated following αPTX3i treatment and the inactivation of PTX3 after bleomycin exposure (Figures 6C and 2H). These results imply that PTX3 is an upstream regulator of CTGF/TGFβ/WNT axis‐mediated fibrotic diseases.

C‐reactive protein (CRP), serum amyloid P (SAP, also known as PTX2) and PTX3 belong to the pentraxin (PTX) family, which has marked structural and functional differences. However, there is no clinical therapeutic strategy targeting CRP in fibrosis. In addition to the acute phase‐responsive CRP, the level of circulating PTX3 is very low in normal human specimens but rapidly increases in the blood of patients with severe inflammation.^13^, ^38^, ^39^ PTX3 is mostly expressed in skeletal and myocardial muscle endothelial cells and stimulated macrophages in inflammatory tissues.^40^ Therefore, it is reasonable to consider that PTX3 acts as a proinflammatory mediator. PTX3 may also be detrimental to the local amplification of inflammation at the site of injury.^41^ Our clinical studies demonstrated that PTX3 signals were strongly detectable in fibroblastic foci of fibrotic lesions but expressed at low levels in the nonfibrotic regions of lung tissues from patients with fILD (Figure 1C), which is consistent with previous studies^14^, ^27^ and demonstrates a close connection between PTX3 levels and pulmonary fibrosis. Notably, PTX2 and PTX3 exert differential effects in fibrosis. PTX2 reduced the differentiation of monocytes towards M2 macrophages and fibrocytes and inhibited lung fibrosis in preclinical models of TGFβ1‐ and bleomycin‐induced fibrosis.^42^, ^43^, ^44^ In contrast, PTX3 promoted fibrocyte differentiation.^16^ The inactivation of PTX3 in our bleomycin‐induced lung injury mouse model attenuated lung injury and fibrosis. Plasma PTX2 levels were not changed in bleomycin‐exposed *Ptx3*‐deficient mice or αPTX3i‐treated mice (Figure S11A,B), which suggests that PTX2 is not involved in PTX3‐regulated pulmonary fibrosis. Notably, our findings indicated that PTX3 was associated with enhanced pulmonary fibrosis and myofibroblast activation, which suggests that PTX3 is involved in ECM production and collagen deposition and contributes to pulmonary fibrosis. In contrast to PTX2, which is produced specifically in the liver in response to inflammatory mediators,^45^ our results imply that the expression of PTX2 was not changed in the plasma of bleomycin‐induced pulmonary fibrosis. However, an increase in plasma Ptx3 was detectable and mimicked the progression of lung injury and fibrosis in response to bleomycin treatment. These data support PTX3 as an ideal diagnostic factor for clinical significance and a therapeutic target for pulmonary fibrosis.

An association between PTX3 and inflammation has been observed in various inflammatory diseases. However, proinflammatory and inflammation‐limiting properties were reported in animal models of diseases. For example, PTX3 plays a role in providing immunity against potential immunological dangers in the lung.^38^ Mice overexpressing PTX3 exhibit enhanced vascular permeability, haemorrhage and neutrophil accumulation in response to ischemia‐reperfusion injury.^46^ PTX3 significantly enhances inflammatory cell infiltration in lung tissue, mucus production and collagen deposition in asthma mouse models.^47^ PTX3 expression in the lung highly correlated with the severity of lung injury in ventilator‐induced lung injury in mice and rats.^48^, ^49^ fILD is also an inflammatory disease. The inflammatory response in the bleomycin‐induced pulmonary fibrosis animal model peaks within the first 7 days and then subsides into a fibrotic phase.^50^ To investigate the anti‐fibrotic effect of anti‐inflammation in response to PTX3 inhibition, we examined αPTX3i treatment and *Ptx3* loss in vivo before bleomycin exposure. The results showed that PTX3 inhibition improved the outcome of lung fibrosis in bleomycin‐induced lung injury and fibrosis (Figures S12 and S13). One study demonstrated that inappropriate therapeutic timing of the experimental fibrosis models is not a viable option except for inducible genetic knockouts.^51^ To mimic the clinical progression from inflammation to fibrosis in fILD patients and evaluate the therapeutic efficiency of PTX3‐based therapy, we investigated αPTX3i therapy and *Ptx3* loss in vivo after bleomycin exposure. Our results suggested that targeting PTX3 significantly recovered body weight loss, reduced the severity of pulmonary fibrosis, and decreased the extent of pulmonary damage in the therapeutic treatment model (Figures 2 and 5). In contrast to our results and proinflammatory and profibrotic claims from the depletion of PTX3 in vivo during bleomycin‐induced pulmonary fibrosis, a distinct experimental approach using systemic *Ptx3*‐knockout mice before bleomycin exposure suggested that PTX3 played an antifibrotic role in pulmonary fibrosis.^52^, ^53^ Consistent with our results that mimicked therapy and anti‐inflammation with induction of *Ptx3* loss and αPTX3i administration (Figures 2 and 5), the dose and timing of PTX3‐based therapy in lung fibrosis must be investigated in the future.

Our recent study showed that CD44 was a direct‐binding PTX3 receptor, and the PTX3/CD44 interaction contributed to the stemness and metastasis of triple‐negative breast cancer cells.^25^ The current study observed induced CD44 expression in bleomycin‐exposed mouse lungs and lung fibroblasts, and our results suggested that fibroblast CD44 mediated PTX3‐induced signalling pathways and consequent fibroblast activation. CD44 expression is responsive to IL‐4, TNF‐α, IL‐1α, and IL‐1β in multiple cell types, including monocytes, epithelial, endothelial, and vascular smooth muscle cells.^54^, ^55^, ^56^ Loss of CD44 attenuated LPS‐ and *Mycobacterium tuberculosis*‐induced macrophage or T lymphocyte migration to the lungs in murine models.^57^, ^58^ CD44 mediates polymicrobial sepsis‐induced pulmonary recruitment of neutrophils.^59^ However, *CD44*‐deficient mice exhibited increased lung inflammation and elevated proinflammatory cytokine release in E. coli‐induced pneumonia and peritonitis, respectively.^60^, ^61^ These studies indicate that CD44 plays a dual role in pro‐ and anti‐inflammation. Therefore, identifying specific ligands for CD44 binding and their signalling pathways and effectors will benefit our understanding of CD44 in inflammatory diseases.

Notably, inducible fibroblast‐specific *Ptx3*‐deficient mice were generated and used to assess the contribution of fibroblast PTX3 to bleomycin‐induced lung injury and fibrosis. As we know, the source of PTX3 is not only from the fibroblasts in the lungs, PTX3 can be produced and released by many cells, such as phagocytes, neutrophils, fibroblasts, and endothelial cells under various stress and inflammatory conditions.^9^ Increased alveolar area and reduced lung fibrosis were observed in bleomycin‐induced systemic *Ptx3*‐deficient mice. Regarding no significant change of plasma PTx3, no improvement in the volume of fibrotic lesions and pulmonary function, which implies the loss of PTX3 in fibroblasts is not sufficient to reduce bleomycin‐induced lung injury. In addition, pirfenidone and nintedanib are approved first‐line antifibrotic therapies for the treatment of IPF and certain fILDs, but their efficacy and side effects indicate large room for improvement.^62^ Our results of αPTX3i safety, therapeutic efficiency in pulmonary fibrosis and compared with pirfenidone and nintedanib support that targeting PTX3 may be an effective strategy for lung injury‐induced fibrosis.

## 4 MATERIALS AND METHODS

### 4.1 Patients and human materials

We obtained plasma specimens from 207 patients with fILD, including 85 patients with IPF, 43 patients with combined pulmonary fibrosis and emphysema, 62 patients with CTD‐related fILD, 15 patients with idiopathic nonspecific interstitial pneumonitis, and two patients with cHP, and 15 subjects without ILD from a cohort that was assembled during two previous research projects investigating the pathogenesis of pulmonary fibrosis between 1 January 2017 and 31 December 2020. The specimens were collected from each patient using an EDTA vacuum collection tube after informed consent was obtained by specialised technicians from the Division of Clinical Pathology, Department of Pathology of National Cheng Kung University Hospital. The specimens were centrifuged at 4°C and 1000× *g* for 20 min. The supernatant was aspirated in a laminar flow bench under aseptic conditions, divided into sterile Eppendorf tubes, and immediately stored at −80°C until use. PTX3 levels in the plasma were measured using ELISA. Pertinent demographic, clinical, and physiological data (including age, sex, body height and weight, Charlson comorbidity index, smoking history, GAP index and stage, serial pulmonary functional parameters, evidence of pulmonary hypertension, and records of acute exacerbation and mortality) were collected from the delinked longitudinal database of the cohort. The research protocols for utilizing human specimens and the delinked clinical database were approved by the Institutional Review Board of National Cheng Kung University Hospital (B‐ER‐110‐092).

### 4.2 Statistical analysis

Categorical data are presented as counts and percentages. Continuous variables are presented as medians and interquartile ranges. No imputation of missing values was made. Variables between patient groups were compared using the Mann–Whitney U test or Kruskal–Wallis test as appropriate, with Bonferroni correction for multiple comparisons. ROC analysis was performed to identify a cut‐off value for the baseline plasma PTX3 level, which was applied to the subsequent Kaplan–Meier and Cox proportional hazard regression analyses to assess correlations between plasma PTX3 levels and clinically important adverse outcomes. In multivariable Cox proportional hazard regression analyses, adjustments were made for covariables, such as age, sex, smoking status, pulmonary hypertension, and the Charlson comorbidity index. All tests were two‐tailed, and a *p* value < .05 was regarded as statistically significant. SPSS (Version 26, SPSS, USA) and GraphPad Prism software (version 5.0) were used for statistical analyses.

### 4.3 Cell culture

The human lung fibroblast cell line HFL1 (BCRC NO. 60299) was obtained from the Bioresource Collection and Research Centre (BCRC, Hsinchu, Taiwan). Ham's F‐12K Kaighn's Medium (Gibco; Thermo Fisher Scientific) was used to culture HFL1 cells supplemented with 10% fetal bovine serum (FBS) (Gibco; Thermo Fisher Scientific), 100 units/ml penicillin and 100 μg/ml streptomycin. The reagents, recombinant protein or antibodies were mixed individually for different experiments: 50 nM wortmannin, 1 μM BAY 11–7085, 7.5 μM JNK inhibitor II, 100 ng/ml PTX3, 200 ng/ml αPTX3i or 200 ng/ml IgG1κ.

### 4.4 Animal model and experimental protocol

Six‐ to eight‐week‐old male C57BL/6J mice were acquired from BioLASCO Taiwan Co. All experiments on mice were based on the guidelines from the Institutional Animal Care and Use Committee (IACUC), NCKU. The animal use protocol was also approved by the IACUC. C57BL/6 mice were exposed to PBS or 2 mg/kg bleomycin (Bleo) (ap302, Znzo) via intratracheal instillation and euthanised at the indicated time points. Administration of αPTX3i (10 mg/kg) (Ohealth Biopharmaceutical (Suzhou) Co., Ltd.) or IgG1κ (10 mg/kg) (10101, Leadgene Biomedical) was performed on days 14 and 21 after bleomycin administration via intraperitoneal injection. Nintedanib (50 mg/kg) or pirfenidone (30 mg/kg) was orally administered twice daily beginning on day 14 and continued until dissection. In the *Ptx3* conditional knockout mice, *Ptx3 ^fl/f^* or *Ptx3^fl/fl^;UBC‐Cre* mice were intratracheally instilled with 2 mg/kg bleomycin. Tamoxifen was administered daily for 5 days using intraperitoneal injection. During the experimental period, body weights were measured every 7 days.

### 4.5 RNA sequencing

Total RNA was isolated from the whole lungs of mice and processed following Illumina's official protocol. For data analysis, sequencing data (FASTQ reads) were generated using Welgene Biotech's pipeline based on Illumina's base calling program bcl2fastq v2.20 and performed using cuffdiff (cufflinks v2.2.1). Functional enrichment assays were performed using clusterProfiler v3.6. Genes with low expression levels (<0.3 FPKM value) in the treated and/or control samples were excluded. Significantly differentially expressed genes were identified when expression levels ≤0.05 and ≥1.5‐fold changed. Gene Ontology (GO) and KEGG pathway analyses were performed using clusterProfiler (R code) for enrichment tests.

### 4.6 Micro‐CT scan of animals

Mice were anaesthetised with 1.5%–2% isoflurane in 100% oxygen on the 28th day after bleomycin administration. Mice were scanned in the supine position using a small‐animal micro‐CT scanner (SkyScan 1076, Belgium) based on a validated scan protocol.^63^ Respiratory monitoring was performed using a pressure transducer pad under the animal's chest. The x‐ray source parameters were 50 kVp combined with a 0.5‐mm aluminium filter, 180 μA current and 120 ms exposure time per projection. A total of 360 views were acquired over a full 360° rotation. The acquisition and rotation time were on the order of 3–5 min per set depending on the respiratory rate and the time needed for rotation to the next angle. X‐ray exposure was triggered once per respiratory cycle in the desired respiratory phase with prospective gating. CT‐Analyzer software (SkyScan; Bruker) was used to segment the foreground from the background to obtain binarised images for each individual lung section. Quantification of the total lung volume and mean lung density was performed for a VOI covering the lung comprised of regions of interest (ROIs) that were manually delineated on the coronal images. Lung images of layers in the coronal plane were defined as follows: layer 1, the level of the carina and layer 2, the level where the inferior vena cava just appeared.

### 4.7 Real‐time quantitative PCR (Q‐PCR)

Total RNA from lung tissues was isolated using TRIsure RNA extraction reagent (Invitrogen). cDNA synthesis was performed using an RT reaction and SuperScript III (Invitrogen). Real‐time quantitative PCR was performed using SensiFAST^™^ SYBR (BIOLINE). Real‐time fluorescence monitoring and melting curve analysis were performed using a CFX Connect Real‐Time PCR System (BIO‐RAD) following the manufacturer's instructions. The following primers were used: mouse Fn1: forward 5′‐ AAGGACAACCGAGGAAACCT ‐3′ and reverse 5′‐ TGTCGCTCACACTTCCACTC‐3′; mouse Col1a1: forward 5′‐ TGGCAAGAATGGAGATGATG‐3′ and reverse 5′‐ ACCATCCAAACCACTGAAGC‐3′; mouse Col1a2: forward 5′‐ TGAAGTGGGTCTTCCAGGTC‐3′ and reverse 5′‐ AGTGAGCCCATTTGTTCCAG‐3′; mouse Col3a1: forward 5′‐ GTTGTGCAATATGCCCACAG‐3′ and reverse 5′‐ GAGACCTGGTTGTCCTGGAA′; mouse Ctgf: forward 5′‐ AAGACACATTTGGCCCAGAC‐3′ and reverse 5′‐ TAGAACAGGCGCTCCACTCT‐3′; mouse Mmp12: forward 5′‐ CCACACTATCCCAGGAGCAT‐3′ and reverse 5′‐ GGTCAAAGACAGCTGCATCA‐3′; mouse Il6: forward 5′‐ CCGGAGAGGAGACTTCACAG‐3′ and reverse 5′‐ TCCACGATTTCCCAGAGAAC‐3′; mouse Tgfb2: forward 5′‐ CGAGGAGTACTACGCCAAGG‐3′ and reverse 5′‐ GGACTGTCTGGAGCAAAAGC‐3′; mouse Ccl2: forward 5′‐CCCAATGAGTAGGCTGGAGA‐3′ and reverse 5′‐ TCTGGACCCATTCCTTCTTG‐3′; mouse Ccn4: forward 5′‐ TGATGATGACGCAAGGAGAC‐3′ and reverse 5′‐ CCTAGGCCACAGGTGGTAGA‐3′; mouse GAPDH: forward 5′‐ TGGTGAAGCAGGCATCTGAG‐3′ and reverse 5′‐ TGAAGTCGCAGGAGACAACC‐3′. Each group was normalised to GAPDH, and results are presented as the difference in fold change.

### 4.8 Measuring the lung hydroxyproline

The right lobes of the lung were weighed and homogenised after the mice were anaesthetised. The lung homogenate was hydrolysed in 12 N HCl overnight at 100°C. The hydroxyproline content of the hydrolysed samples was assessed by the absorbance at 560 nm with a hydroxyproline assay kit (# 6017; Chondrex Inc.) according to the manufacturer's recommendations. The absorbance of each sample was determined using an ELISA plate reader at 560 nm.

### 4.9 Data and code availability

The access number for the RNA sequencing (RNA‐Seq) data reported in this paper is [GEO]: GSE210165 (https://www.ncbi.nlm.nih.gov/geo/query/acc.cgi?acc = GSE210165).

### 4.10 Western blot analysis

Cells were harvested and homogenised in modified radioimmunoprecipitation assay buffer (modified RIPA) supplemented with 50 mM Tris‐HCl (pH 7.4), 1% NP‐40, 0.25% sodium deoxycholate, 150 mM NaCl, 1 mM EDTA, 1 mM dithiothreitol (DTT), aprotinin (1 mg/ml), 1 mM phenylmethylsulphonyl fluoride and leupeptin (1 mg/ml). Primary antibodies against fibronectin (#15613‐1‐AP, ProteinTech), Collagen I (#14695‐1‐AP, ProteinTech), α‐SMA (GTX100904, GeneTex), phospho‐SAPK/JNK (Thr183/Tyr185) (#4668; Cell Signaling), SAPK/JNK (#9252; Cell Signaling), phospho‐NF‐κb p65 (Ser536) (#3033; Cell Signaling), NF‐κb p65 (GTX107678; GeneTex), phospho‐c‐Jun (Ser 63) (#2361, Cell Signaling), c‐Jun (#9165, Cell Signaling), phospho‐p44/42 MAPK (ERK1/2) (Thr202/Tyr204) (#4377; Cell Signaling), p44/42 MAPK (ERK1/2) (#9102; Cell Signaling), CD44 (ab157107; Abcam) and α‐tubulin (T6199, Sigma) were used for Western blotting. α‐Tubulin was used as the loading control.

### 4.11 Proximity ligation assay

HFL1 cells were seeded on coverslips and treated with or without 100 ng/ml His‐PTX3 fusion protein for 1 h. The cell membrane was stained with PKH67, and 4% paraformaldehyde was used to fix the cells. PLA was performed based on the manufacturer's instructions (Sigma). Two different primary antibodies were used to recognise His‐PTX3 and the active form of CD44. The secondary antibodies are known as PLA probes (one PLUS and one MINUS). The protein interactions were observed as different bright red spots and assessed using fluorescence microscopy. Randomly selected fields of view were photographed at a magnification of ×1000. Protein interactions were quantified by calculating the number of spots per cell.

### 4.12 Isolation of mouse primary lung fibroblasts

Six‐ to eight‐week‐old male C57BL/6J mice (BioLASCO, Taiwan Co) were used for normal primary mouse lung fibroblast isolation. Mouse lungs were washed with PBS and minced using sterile scissors. The minced tissue was incubated with trypsin‐EDTA buffer (0.25%) on a shaker at 37°C for 30 min. The digested tissue was neutralised with DMEM, filtered with a 70‐μm cell strainer (Corning Falcon) and seeded into culture dishes. The cells were cultured in DMEM containing 10% FBS (Gibco; Thermo Fisher Scientific), 100 mg/ml streptomycin and 100 U/ml penicillin at 37°C in a 5% CO_2_ incubator. Upon reaching the desired percentage of cellular confluence, the cells were trypsinised and subcultured until use in subsequent experiments. Mouse lung fibroblasts were generated using cells between passages 2 and 6.

### 4.13 MTT assay

To evaluate the viability of fibroblasts in response to αPTX3i, HFL1 cells were incubated with different concentrations of αPTX3i for 24, 48 and 72 h. To assess the proliferation of fibroblasts, HFL1 cells were treated with different concentrations of PTX3 recombinant protein for 24, 48 and 72 h. Experimental cells were incubated with MTT [3‐(4,5‐dimethylthiazol‐2)‐2,5‐diphenyltetrazolium bromide] (M5655, Sigma) for 3 h. After discarding the supernatant, dimethyl sulphoxide (DMSO) was added to each well and mixed on a plate shaker for 10 min at room temperature. An ELISA plate reader was used to quantify the absorbance of each sample at 490 nm.

### 4.14 Immunofluorescence, confocal microscopy and image analysis

HFL1 cells were grown on coverslips and treated with or without 100 ng/ml His‐PTX3 fusion protein for 24 h. Cells were fixed in 4% paraformaldehyde for 20 min and incubated with antibodies against His (sc‐803, Santa Cruz) and CD44 (GTX83114, GeneTex). For lung sections, samples were incubated with PTX3 (1:100, ab90806; Abcam), fibronectin (1:350 dilution, #15613‐1‐AP, ProteinTech) and α‐SMA (1:100 dilution, GTX100904, GeneTex) at room temperature for 1 h. Samples were incubated with Alexa 568‐ or 488‐conjugated secondary antibodies (Invitrogen) at a 1:200 dilution. The samples were washed with 0.1% Tween 20 in PBS and stained with DAPI (P36935, Invitrogen) on coverslips. The fluorophores were excited by a laser at 405, 488, or 543 nm and observed using an FV‐3000 confocal system (Olympus). Randomly chosen fields were photographed at ×200 magnification under a fluorescence microscope.

### 4.15 Immunohistochemistry

Lung sections were fixed in 4% paraformaldehyde for 10 min, embedded in paraffin and cut into 4‐mm thick sections. For histological examination, lung sections were stained with H&E, Sirius red (ab246832, Abcam) or Masson's trichrome (Carl Roth GmbH, Karlsruhe, Germany). The level of fibrosis was quantified in accordance with Sirius red and Masson's trichrome staining. Immunohistochemical staining for PTX3 (1:100, ab90806; Abcam) was performed. First, sections were deparaffinised with xylene, dehydrated with ethanol, and heated in 10 mM citrate buffer (pH 6.0) for antigen retrieval. Sections were incubated in 3% H_2_O_2_ for 5 min at room temperature to inactive endogenous peroxidase activity and then blocked in a blocking buffer. Sections were incubated with αPTX3 antibodies for 1 h at room temperature and incubated with secondary antibody and antibody‐coated polymer peroxidase complexes (RE7290‐K, Leica) for another 3 min at room temperature. DAB chromogen (RE7290‐K, Leica) was used for further incubation for 5 min at room temperature. Sections were stained with haematoxylin for 10 s and washed for 5 min in running water. Sections were viewed and imaged using a laser scanning confocal system consisting of a BX51 microscope (Olympus), a DP70 digital camera system and DP Controller software (Olympus). Randomly chosen fields were photographed at ×200 magnification under a fluorescence microscope. To quantitate the Immunohistochemistry (IHC) staining intensity, the percentage of IHC signal per imaged field was analysed using ImageJ software.

### 4.16 Pulmonary function assessment

Whole‐body plethysmography (WBP; Buxco Research Systems, Wilmington, NC) was used to assess lung function. For invasive measurement, mice were anaesthetised with Zoletil/Rompun solution, and tracheal cannulas were inserted. Mice were placed in a resistance/compliance (RC) collection station (FinePointe RC; Buxco Research Systems, Winchester, UK) and mechanically ventilated at a rate of 150 breaths/min. PBS was administered via the trachea, and changes in minute volume (MV), dynamic compliance (Cdyn), static lung compliance (Cstatic) and lung resistance (Rl) are expressed as a mean value of 3 min of recording.

### 4.17 Assessment of liver and renal function

C57BL/6J mice were intraperitoneally injected with IgGκ1 (10 mg/kg) or αPTX3i (10 mg/kg) daily for 42 days. Liver and renal functions were assessed by measuring blood alkaline phosphatase (ALP), aspartate aminotransferase (GOT/AST), alanine aminotransferase (GPT/ALT), urea and serum creatinine (CRE) at the Medical Laboratory of Yang‐Ming.

### 4.18 Migration assays

HFL1 cells were seeded in 24‐well plates containing 8‐μm pore inserts (353097; BD Biosciences). The experimental medium contained 50 nM wortmannin, 1 μM BAY 11–7085 or 7.5 μM JNK inhibitor II in the absence or presence of 100 ng/ml PTX3 and was placed in the lower wells. DMEM with 10% FBS was added to the upper wells of the 24‐well plates. The remaining cells that had not migrated inside the insert were removed using cotton swabs after 16 h of incubation. Cells that migrated to the bottom of the membrane were stained with DAPI. The total number of cells attached to the lower surface of the polycarbonate filter insert was determined at 200× magnification under a fluorescence microscope.

### 4.19 Measurement of PTX3 and PTX2 detection in plasma

The concentrations of Ptx3 and Ptx2 in the plasma were determined using Human PTX3 (DPTX30, R&D Systems), mouse Ptx3 (MPTX30, R&D Systems) and mouse Ptx2 (MPTX20, R&D Systems) ELISA kits following the manufacturer's instructions. Samples or reference standards (50 μl) were added to each well of a microplate coated with 50 μl biotinylated antibody specific to PTX3 or PTX2 and incubated for 120 min at room temperature. A horseradish peroxidase (HRP)‐conjugated polyclonal secondary antibody was added to the wells (100 μl/well) after washing unbound proteins and incubated for 120 min at room temperature in the dark. After washing with the washing reagent, 100 μl tetramethyl benzidine (TMB) was added to the wells for 15 min at room temperature in the dark. Finally, 100 μl termination solution was added to the wells, and the optical density was determined immediately at 450 nm. The data were quantified using the linear regression equation of the standard curve.

### 4.20 ELISA

The wells of the plates (9018; Corning Costar) were coated with 10 μg/ml CD44 in phosphate‐buffered saline (PBS) at pH 7.2 overnight at 4°C and blocked with a blocking buffer containing 3% BSA in PBS for 1 h at room temperature. HRP‐conjugated PTX3 (32310; LEADGENE) was diluted to the desired concentration in PBS for the binding experiments. These solutions were added to the wells and incubated for 2 h at room temperature. After washing three times with PBS, the bound HRP conjugate was detected using TMB. After 15 min, the peroxidase reaction was stopped by the addition of 0.1 M H_2_SO_4_. The optical densities at 450 nm were measured using an ELISA reader.

### 4.21 Picrosirius red staining and spectrophotometric analysis

Following the protocol mentioned in a previous study,^27^ HFL1 cells were seeded in 24‐well plates and treated with or without 100 ng/ml PTX3. Experimental cells were fixed with methanol at −20°C overnight. Cells were stained with Picrosirius Red Solution (ab246832, Abcam) for 1 h at room temperature and rinsed with an acetic acid solution. Quantification of the total number of nodule foci was determined visually under a microscope.

### 4.22 Statistics

RNA‐seq data were analysed using Illumina's official protocol and are described in detail in the Supporting Information and Methods. GraphPad Prism software (version 5.0) was used for all other analyses. Statistical analyses were performed using two‐sided Student's *t*‐tests or one‐way ANOVA as indicated in the figure legends. *p* values were corrected for multiple hypothesis testing using Dunnett's test (for comparisons between multiple treatments and a single control). *p* values <.05 were considered statistically significant.

### 4.23 Study approval

Patient plasma was obtained under the auspices of the National Cheng Kung University Hospital Institutional Review Board–approved protocol (B‐ER‐110‐092). Following the observation that PTX3 was increased in human fILD, we used a bleomycin‐induced lung fibrosis animal model to verify and mimic the above observation and established a platform for further investigation and validation of PTX3 inhibitors. Animal procedures were performed according to the protocols approved by the IACUC of National Cheng Kung University (approval 109190 and 111240).

## CONFLICT OF INTEREST

The authors declare no conflict of interest.

## Supporting information

Supporting Information
