# Supplementary material for: Blockade of the pentraxin 3/CD44 interaction attenuates lung injury‐induced fibrosis
Source: Clin Transl Med. 2022 Nov 6;12(11):e1099. doi: 10.1002/ctm2.1099 (PMC9637652; doi:10.1002/ctm2.1099)
Supplement: Supplementary file 1 — Supporting Information [file CTM2-12-e1099-s001.docx]

**Supplementary Materials**

Fig. S1. PTX3 expression in the lungs of bleomycin-exposed mice.

Fig. S2. The effect of bleomycin-induced pulmonary fibrosis in inducible fibroblast Ptx3-deficient mice.

Fig. S3. PTX3 activates expression of fibronectin, collagen I and α-SMA, but has no effect on cell proliferation.

Fig. S4. Fibrotic markers in response to the interaction of PTX3 and CD44.

Fig. S5. PTX3 activates AKT1, JNK, c-Jun and NF-κB (p65) in HFL1 cells and primary mouse lung fibroblasts.

Fig. S6. Signaling pathways and consequent effectors in response to the PTX3/CD44 interaction in HFL1 cells and primary mouse lung fibroblasts.

Fig. S7. Safety and toxicity assessment of αPTX3i in mice and fibroblasts, respectively.

Fig. S8. αPTX3i attenuates bleomycin-induced pulmonary fibrosis in a dose-dependent manner.

Fig. S9. The level of hydroxyproline was increased in lung tissue of bleomycin-exposed mice.

Fig. S10. αPTX3i inhibites PTX3/CD44-induced fibrotic markers and signaling pathways.

Fig. S11. The response of PTX2 in PTX3-regulated pulmonary fibrosis.

Fig. S12. Bleomycin-induced pulmonary fibrosis is attenuated in conditional *Ptx3* pre-inactivated mice.

Fig. S13. Preventative αPTX3i treatment significantly attenuates bleomycin-induced pulmonary fibrosis in mice.

Table S1. Baseline characteristics of the 222 enrolled subjects.

Table S2. Comparison of baseline plasma levels of pentraxin 3 among different subgroups as stratified according to age and disease severity.

**Fig. S1. PTX3 expression in the lungs of bleomycin-exposed mice.**


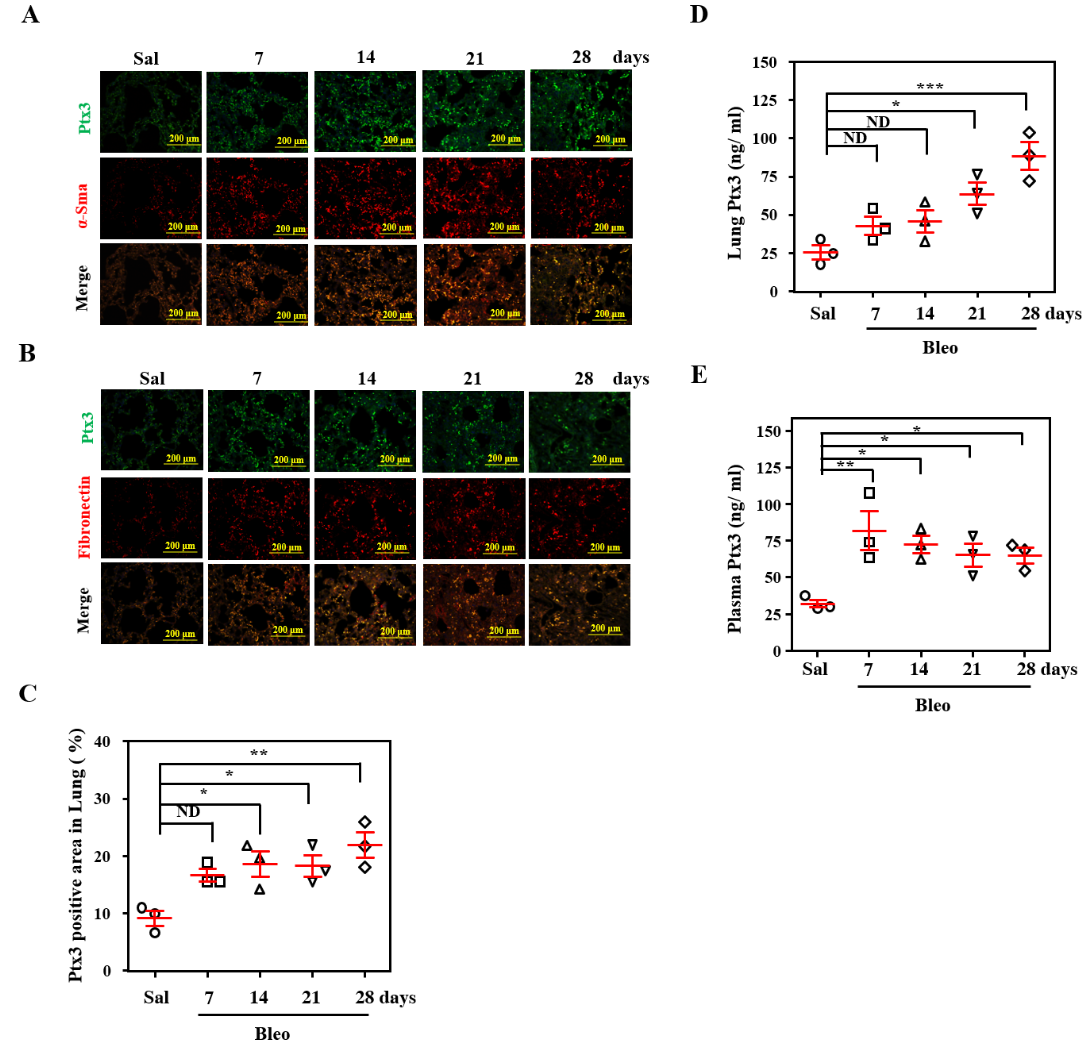


C57BL/6 mice were intratracheally instilled with PBS or 2 mg/kg bleomycin (Bleo) on day 0 and euthanized on day 7, 14, 21, 28 (n = 3, each group). **(A-B)** Representative immunostaining of lung sections for Ptx3, α-Sma, and Fibronectin. Scale bars are 200 μm. **(C)** Quantification of Ptx3-positive area normalized to DAPI-positive area in lung sections. **(D)** Lung tissue Ptx3 and **(E)** Plasma Ptx3 concentrations were measured in bleomycin-exposed mice on days 0, 7, 14, 21, and 28 by ELISA. All data are shown as the mean ± SEM. Differences among the groups were analyzed using one-way ANOVA followed by Tukey’s multiple comparison test. *p < 0.05, **p < 0.01, ***p < 0.001, ND: no difference.

**Fig. S2. The effect of bleomycin-induced pulmonary fibrosis in inducible fibroblast Ptx3-deficient mice.**


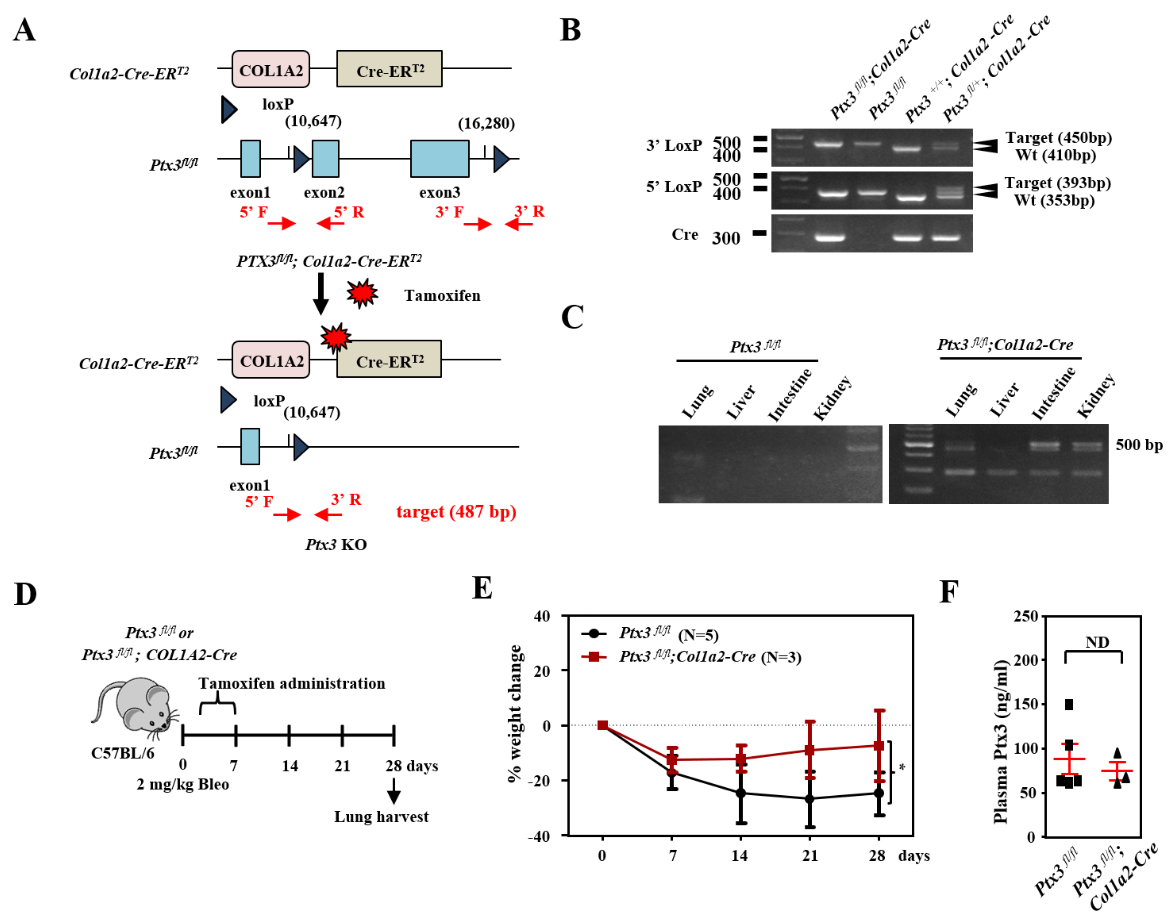


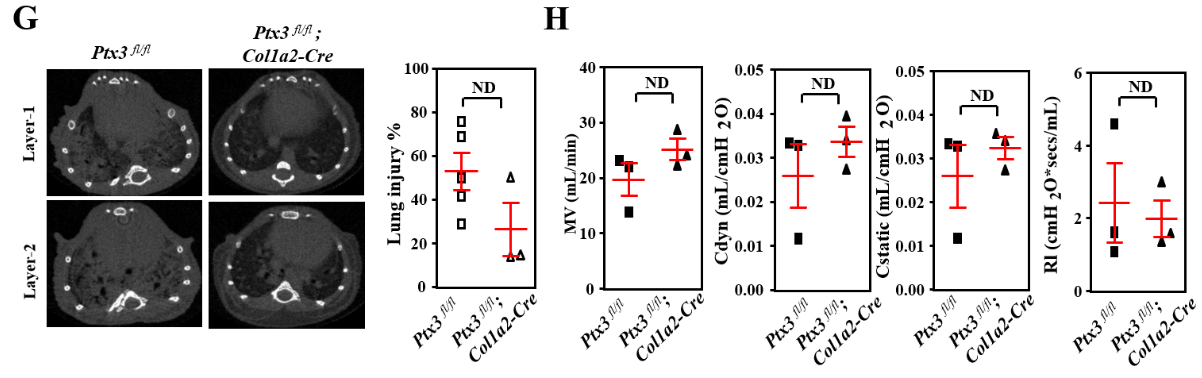


**(A)** Schematic representation of the inducible fibroblast-restricted *Ptx3* knockout mouse model. *Col1a2-Cre-ERT2* transgenic mice were crossed with wild-type (*Ptx3 ^fl/fl^*) mice (n=5) to produce tamoxifen-inducible fibroblast-specific *Ptx3* knockout (*Ptx3 ^fl/fl^*; *Col1a2-Cre)* mice (n=3). **(B)** *Ptx3 ^fl/fl^*; *Col1a2-Cre-ERT2,* in which *Ptx3* is deleted in widespread cells/tissues. Representative genotype PCR analysis of genomic DNA from tail tissues of *Ptx3 ^fl/fl^*; *Col1a2-Cre-ERT2*, in which “fl” represents floxed and “+” represents wild type. PCR genotyping of mice for *Ptx3* and Cre. Tail DNA was amplified using primers 5’ loxP site, generating products of 353 bp and 393 bp for the WT and Target, and using primers 3’ loxP site, generating products of 410 bp and 450 bp for the WT and Target, respectively. For Cre genotyping, Cre-specific primers generate a band of 288 bp. **(C)** PCR diagnostic for activated ERT2-Cre-mediated knockout of exons 2 and 3 of the *Ptx3* allele in genomic DNA isolated from lung, liver, intestine and kidney of *Ptx3 ^fl/fl^* or *Ptx3 ^fl/fl^*; *Col1a2-Cre-ERT2* mice treated with tamoxifen for 5 days. **(D)** Scheme showing the experimental setup. *Ptx3 ^fl/fl^* mice and *Ptx3 ^fl/fl^*; *Col1a2-Cre-ERT2* mice were intratracheally instilled with 2 mg/kg bleomycin (Bleo) on day 0 and euthanized on day 28. Tamoxifen was administered daily from days 3 to 7 by intraperitoneal injection. **(E)** Body weight was measured for 28 days post bleomycin administration as a percentage of day 0 weight for each mouse group as indicated. **(F)** Plasma Ptx3 concentrations were measured in *Ptx3 ^fl/fl^* and *Ptx3 ^fl/fl^*; *Col1a2-Cre-ERT2* mice on day 28 post-bleomycin administration. **(G)** Representative computed tomography slices of mouse lungs on day 28 post bleomycin administration. Quantitative analysis of lung injury was performed on micro-CT sections using CT-Analyzer software. **(H)** Total respiratory system minute volume (MV), dynamic compliance (Cdyn), static lung compliance (Cstatic) and lung resistance (Rl) were measured on day 28 post bleomycin administration. All data are shown as the mean ± SEM. Differences among the groups were analyzed using unpaired two-tailed t tests. *p < 0.05, ND: no difference.

**Fig. S3. PTX3 activates expression of fibronectin, collagen I and α-SMA, but has no effect on cell proliferation.**


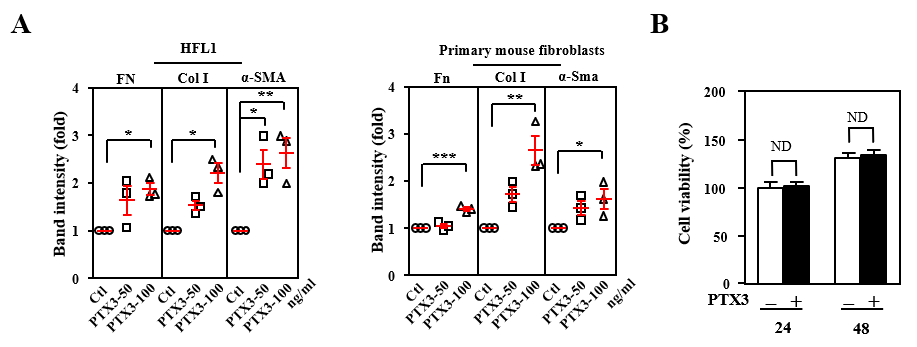


**(A)** Human lung fibroblasts (HFL1) or primary mouse lung fibroblasts were treated with or without 50 ng/ml or 100 ng/ml PTX3 for 6 h. Quantitative analysis of the levels of Fibronectin (Fn), Collagen I (ColI) and α-SMA (α-Sma) in PTX3-treated HFL1 cells and primary mouse lung fibroblasts. Immunoblotting was replicated independently at least three times per experiment. **(B)** HFL1 cells were incubated with recombinant PTX3 as indicated for 24 h and 48 h. Cell viability was determined by MTT assay. All data are expressed as the mean ± SEM. Differences among the groups were analyzed using unpaired two-tailed t tests. *p < 0.05, **p < 0.01, ***p < 0.001, ND: no difference.

**Fig. S4. Fibrotic markers in response to the interaction of PTX3 and CD44.**


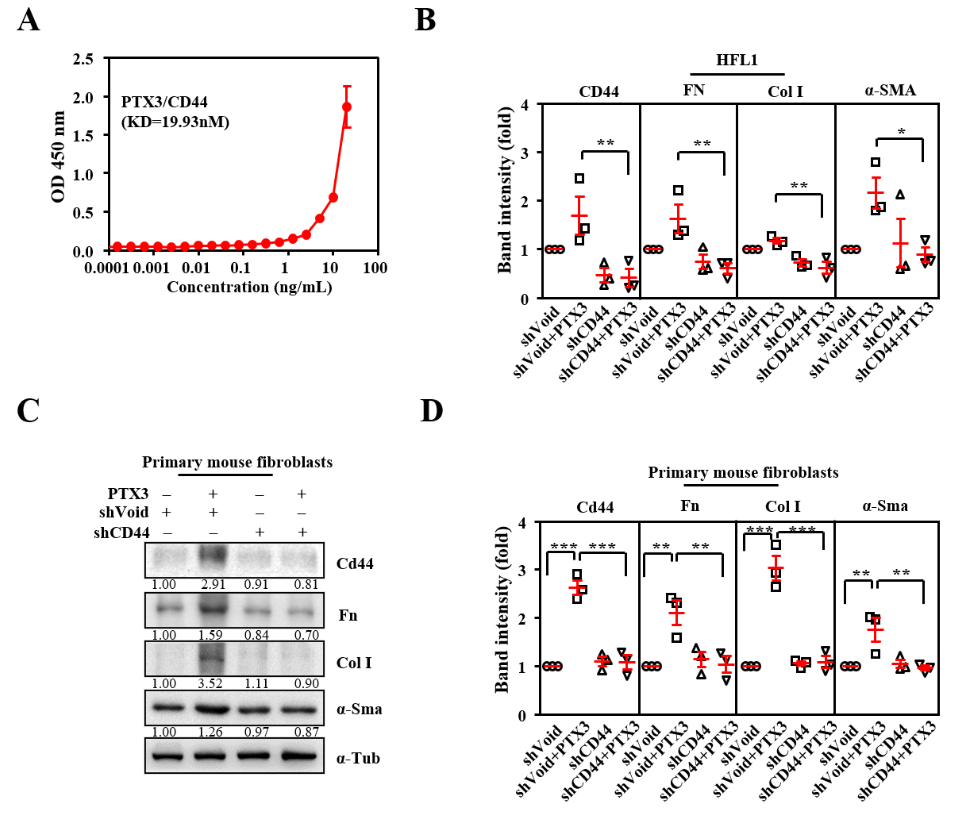


**(A)** The CD44 binding ability of PTX3 was assessed by incubation of recombinant PTX3 and CD44 proteins, and their interaction was analyzed by ELISA. **(B)** HFL1 cells or **(C)(D)** primary mouse lung fibroblasts were infected with control (shVoid) and shCD44 lentiviruses and then treated with PTX3 for 6 h. Quantitative analysis of the levels of CD44, fibronectin (Fn), Collagen I (ColI) and α-SMA (α-Sma) in PTX3-treated cells. The immunoblotting was replicated independently at least three times per experiment. All data are shown as the mean ± SEM. Differences among the groups were analyzed using unpaired two-tailed t tests. *p < 0.05, **p < 0.01, ***p < 0.001.

**Fig. S5. PTX3 activates AKT1, JNK, c-Jun and NF-κB (p65) in HFL1 cells and primary mouse lung fibroblasts.**


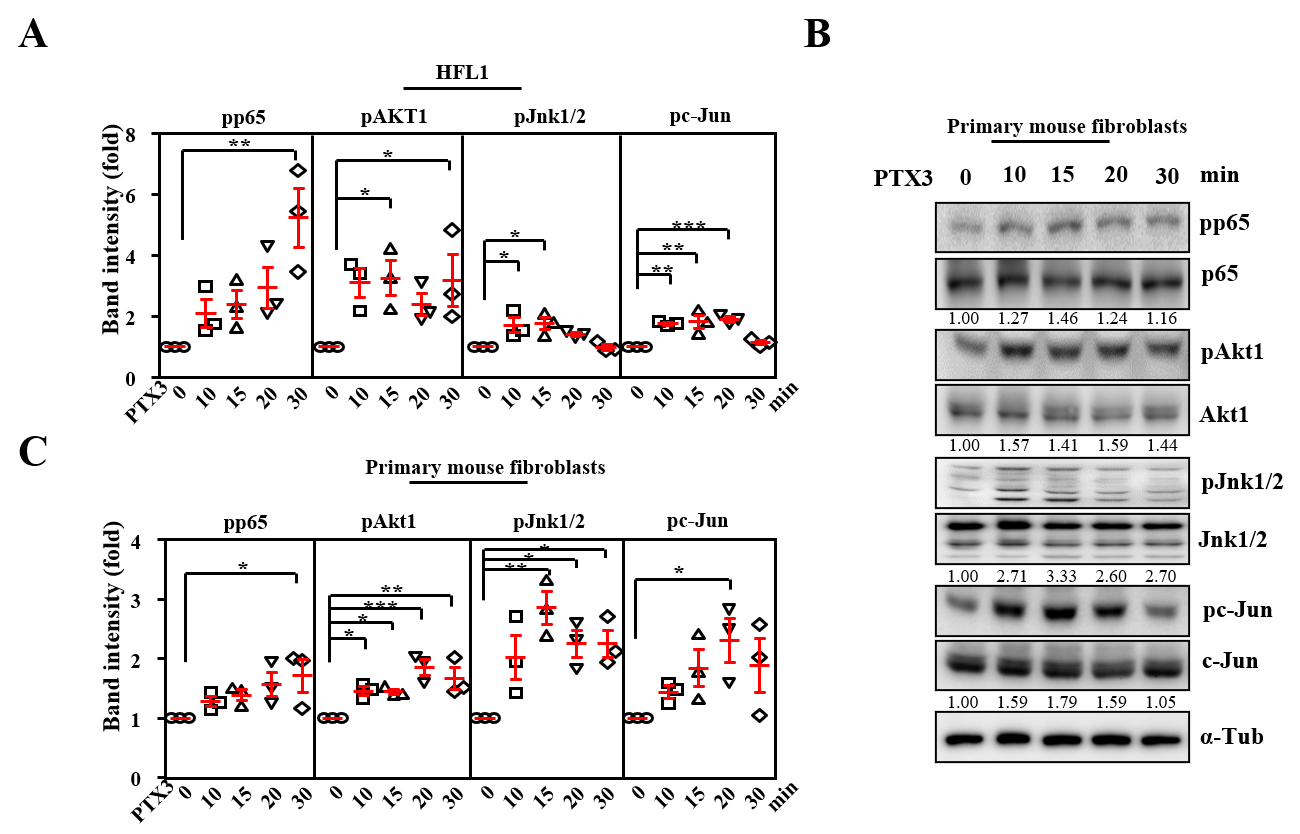


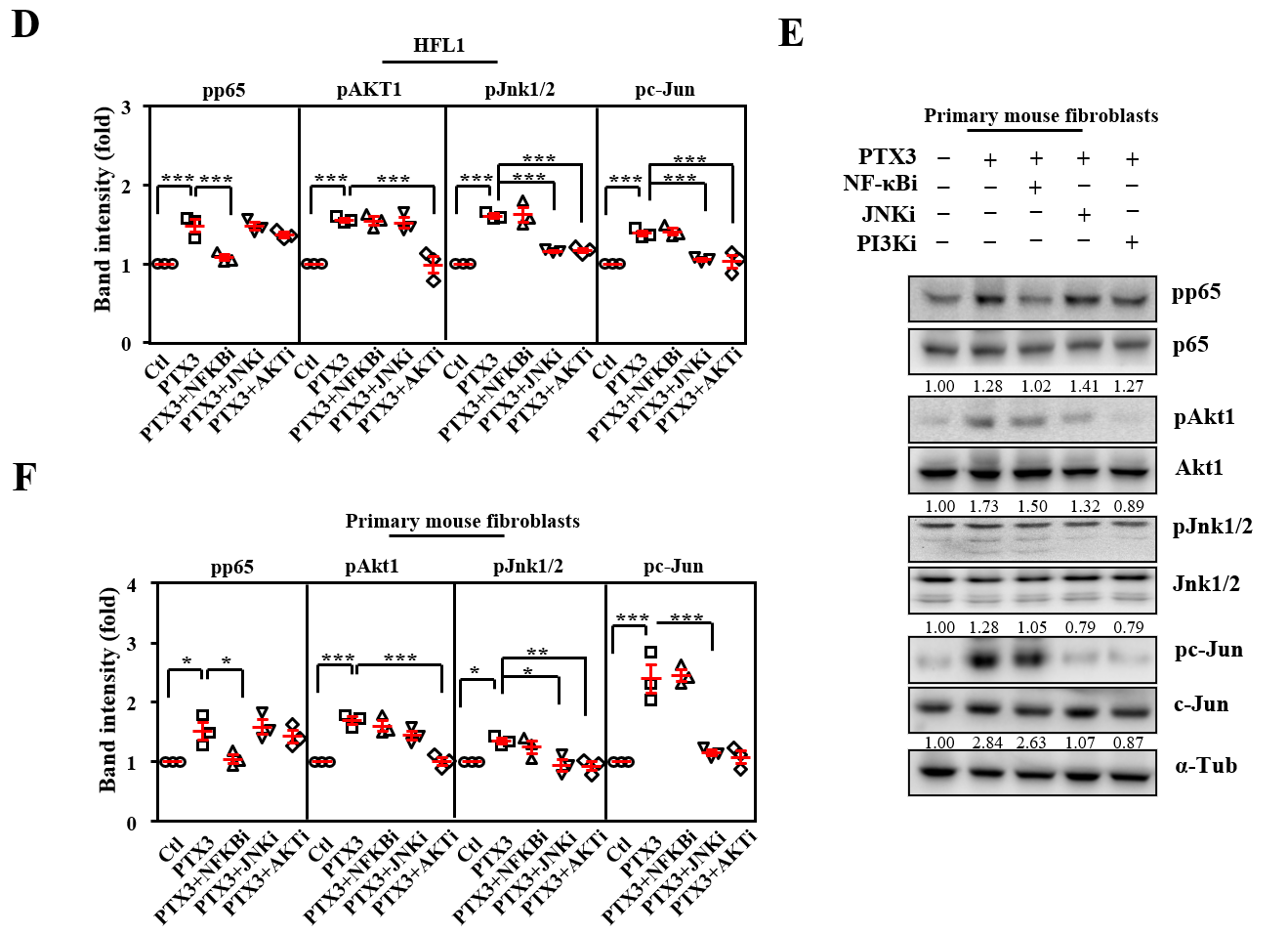


**(A)** HFL1 cells or **(B)(C)** primary mouse lung fibroblasts were treated with PTX3. The cell lysates were harvested as indicated time and further performed with Western blot. Quantitative analysis of the levels of pAKT1, pJNK, pc-Jun and pp65 in cells. **(D)** HFL1 cells or **(E)(F)** primary mouse lung fibroblasts were pretreated with or without wortmannin (50 nM), BAY 11-7085 (1 µM) or JNK inhibitor II (7.5 µM) and then treated with PTX3. Quantitative analysis of the activation of pAKT1, pJNK, pc-Jun and pp65 with their individual protein abundance in the images of Western blot. Immunoblotting was replicated independently at least three times per experiment. All data are shown as the mean ± SEM. Differences among the groups were analyzed using unpaired two-tailed t tests. *p < 0.05, **p < 0.01, ***p < 0.001.

**Fig. S6. Signaling pathways and consequent effectors in response to the PTX3/CD44 interaction in HFL1 cells and primary mouse lung fibroblasts.**


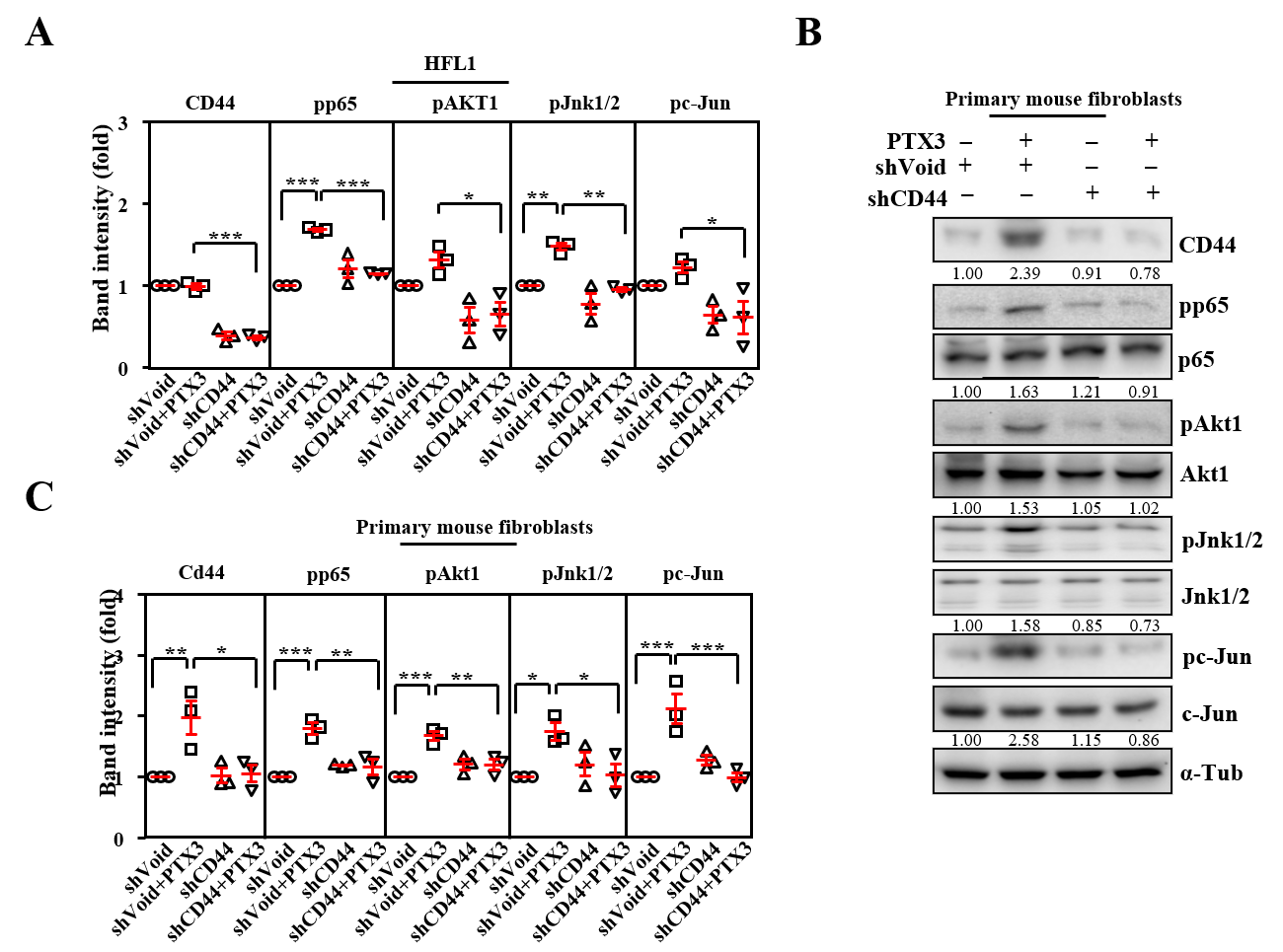


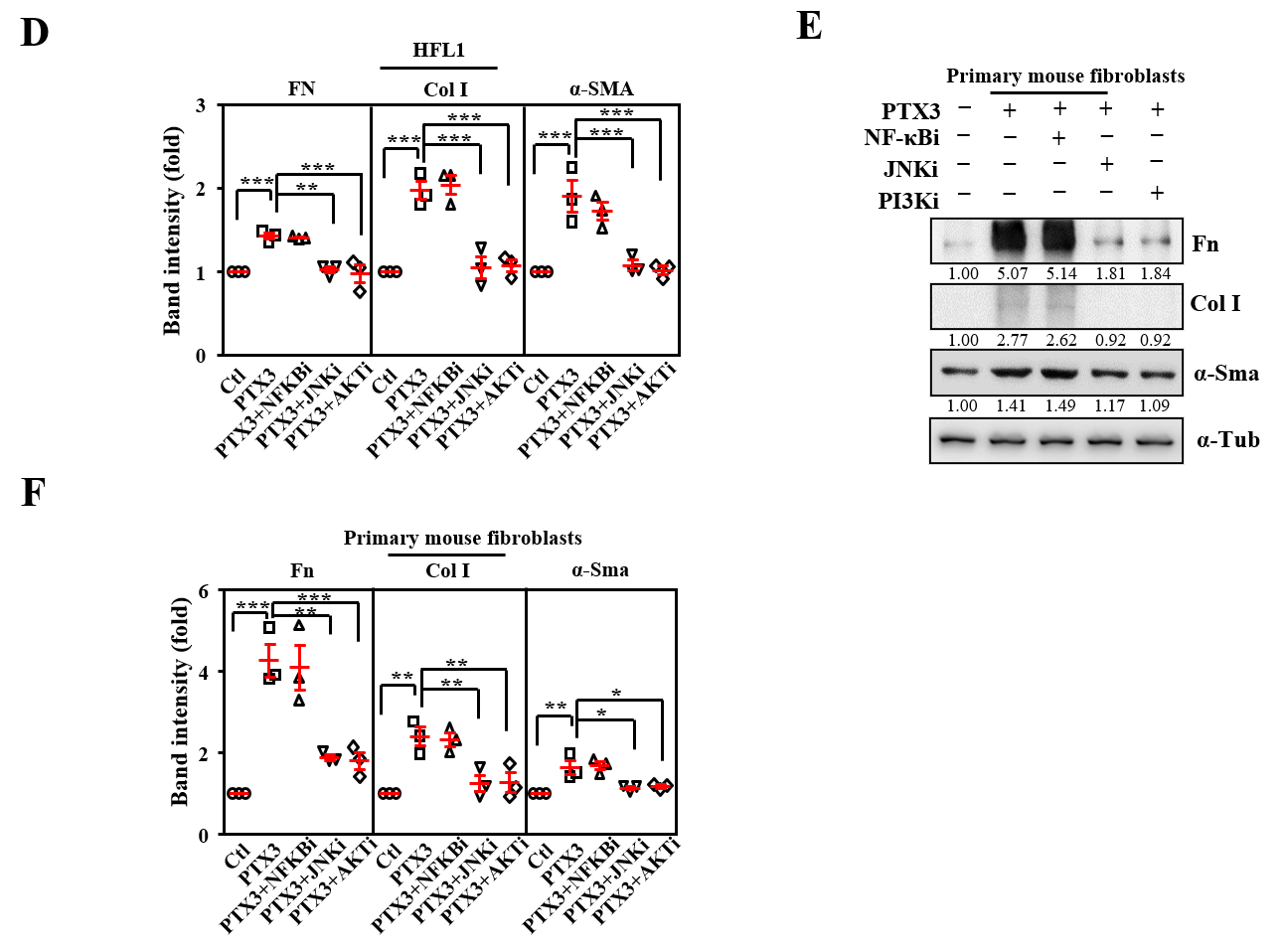


**(A)** HFL1 cells or **(B)(C)** primary mouse lung fibroblasts were infected with lentiviral shVoid and shCD44 and then treated with PTX3. Quantitative analysis of the activation of pAKT1, pJNK, pc-Jun and pp65 with their individual protein abundance in the images of Western blot. **(D)** HFL1 cells or **(E)(F)** primary mouse lung fibroblasts were pretreated with indicated inhibitors before PTX3 treatment. Quantitative analysis of the activation of fibronectin (Fn), Collagen I (ColI) and α-SMA (α-Sma) abundance in the images of Western blot. Immunoblotting was replicated independently at least three times per experiment. All data are shown as the mean ± SEM. Differences among the groups were analyzed using unpaired two-tailed t tests. *p < 0.05, **p < 0.01, ***p < 0.001.

**Fig. S7.** **Safety and toxicity assessment of αPTX3i in mice and fibroblasts, respectively.**


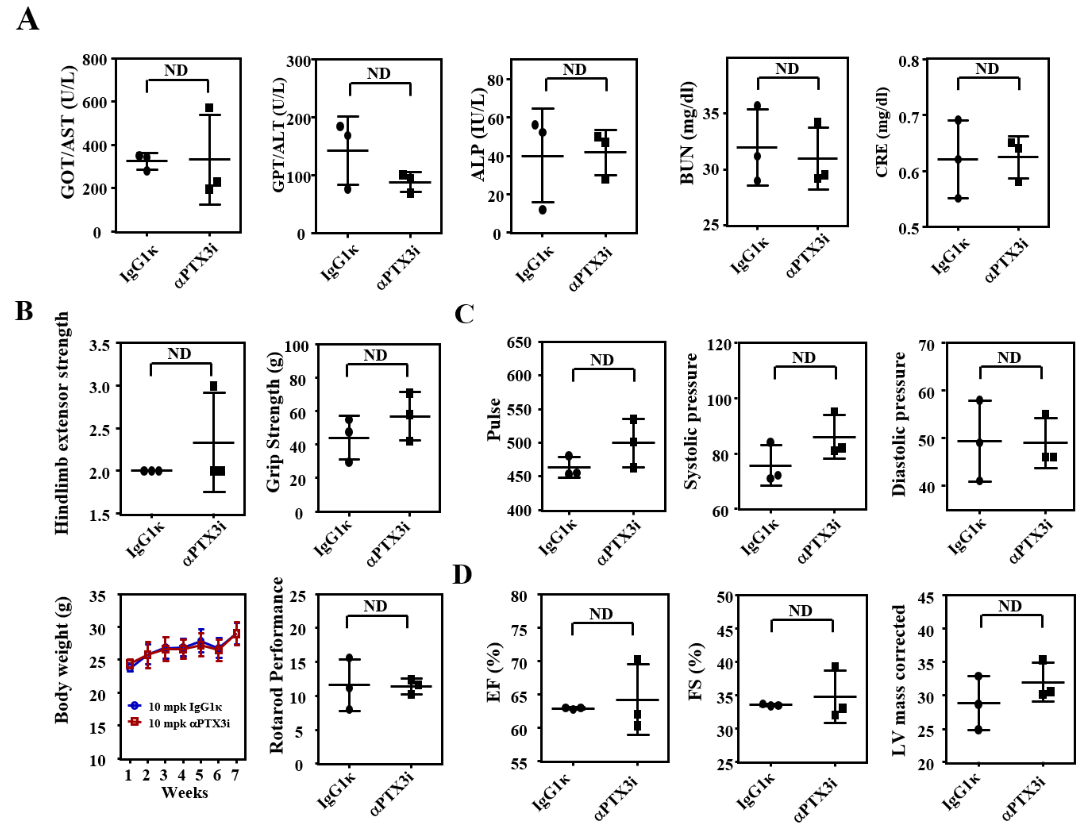


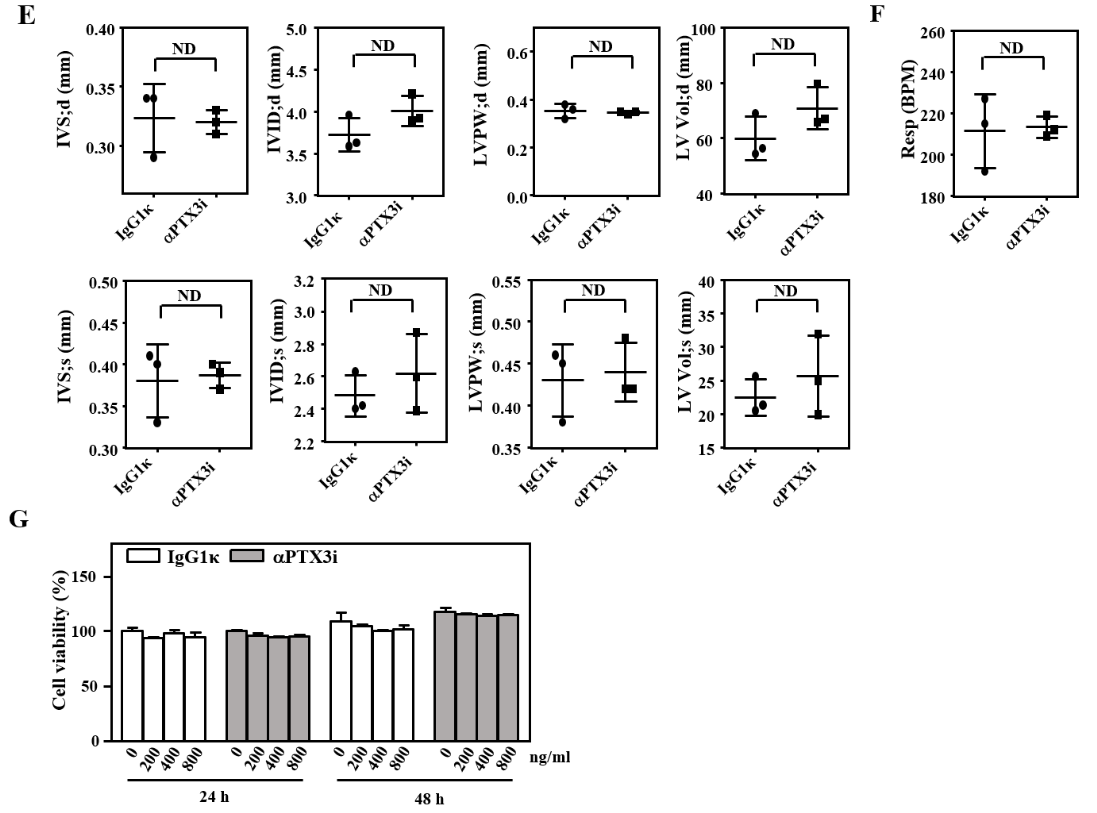


The toxicity and safety assessments were examined using **(A)** indicated liver and renal index factors in plasma**.** **(B)** Neuromuscular and physiological observations of the central nervous system. **(C)** Physiological parameters were examined via blood pressure. **(D)** Cardiac function was assessed by ejection fraction (EF), fractional shortening (FS), left ventricular mass corrected (LVMc) and **(E)** left ventricular internal diameter, diastole (LVIDd), left ventricular internal diameter, systole (LVIDs), interventricular septal thickness in diastole (IVSd), interventricular septal thickness in systole (IVSs), left ventricular end-systolic posterior wall-depth (LVPWd), left ventricular end-systolic posterior wall thickness (LVPWs), left ventricular end-diastolic volume (LV Vol d), left ventricular end-systolic volume (LV Vol s). **(F)** Respiratory rate in breaths per minute (BPM) in day 42 of IgG1κ-treated and αPTX3i-treated mice. **(G)** HFL1 cells were incubated with the indicated concentrations of IgG1κ and αPTX3i for 24 h and 48 h. Viable cell numbers were determined using the MTT method. All data are shown as the mean ± SEM. Differences among the groups were analyzed using unpaired two-tailed t tests. ND: no difference.

**Fig. S8.** **αPTX3i attenuates bleomycin-induced pulmonary fibrosis in a dose-dependent manner.**


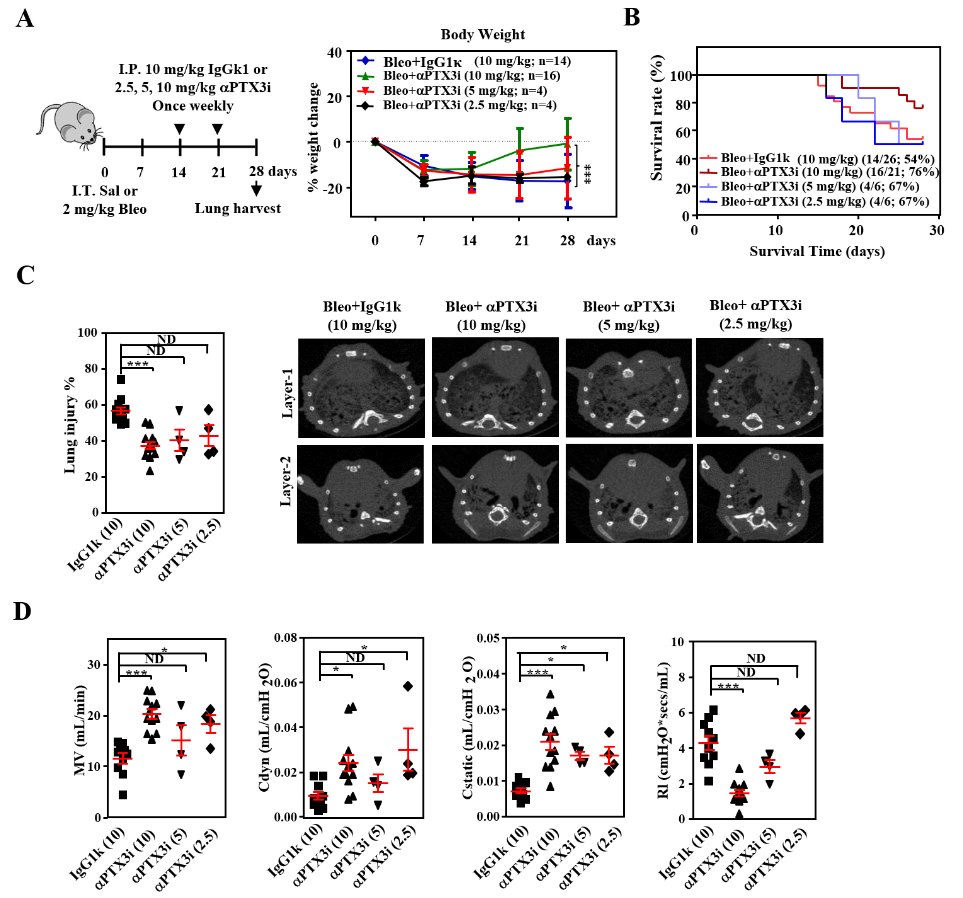


**(A)** Scheme showing the experimental flowchart. C57BL/6 mice were intratracheally instilled with 2 mg/kg bleomycin (Bleo) on day 0 and euthanized on day 28. IgG1κ (10 mg/kg) or αPTX3i (2.5, 5, 10 mg/kg) was administered on days 14 and 21 by intraperitoneal injection. Body weight was continually measured for 28 days post bleomycin administration as a percentage of day 0 weight for each group as indicated. (Bleo + IgG1κ (10 mg/kg): n=14, Bleo+αPTX3i (2.5 mg/kg): n=16, Bleo+αPTX3i (5 mg/kg): n=4, Bleo+αPTX3i (10 mg/kg): n=4). **(B)** Representative computed tomography slices of mouse lungs on day 28 from the IgG1κ and αPTX3i groups following bleomycin administration. Quantitative analysis of lung injury was examined on the micro-CT sections using CT-Analyzer software. **(C)** Minute volume (MV), dynamic compliance (Cdyn), static lung compliance (Cstatic) and lung resistance (Rl) were measured on day 28 experimental animals. **(D)** Percentages of surviving mice over the 28-day period in the IgG1κ (10 mg/kg), αPTX3i (10 mg/kg), αPTX3i (5 mg/kg) and αPTX3i (2.5 mg/kg) groups were plotted. All data are shown as the mean ± SEM. Differences among the groups were analyzed using one-way ANOVA followed by Tukey’s multiple comparison test. *p < 0.05, **p < 0.01, ***p < 0.001, ND: no difference.

**Fig. S9. The level of hydroxyproline was increased in lung tissue of bleomycin-exposed mice.**


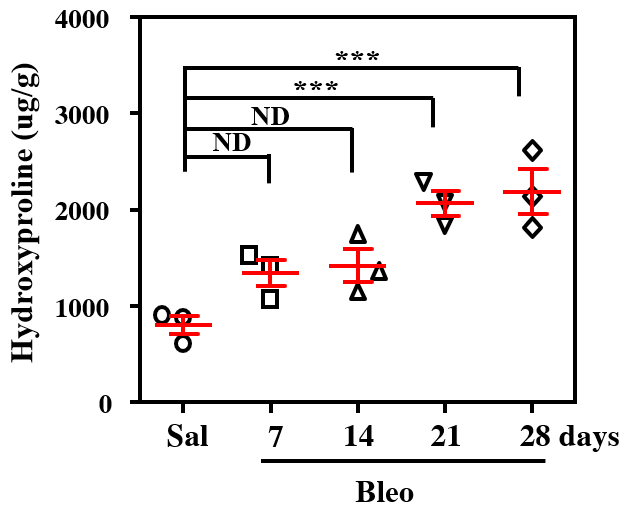


C57BL/6 mice were intratracheally instilled with PBS or 2 mg/kg bleomycin (Bleo) on day 0 and euthanized on day 7, 14, 21, 28 (n = 3, each group). Lung hydroxyproline concentrations were measured using a hydroxyproline assay kit.

**Fig. S10. αPTX3i inhibits PTX3/CD44-induced fibrotic markers and signaling pathways.**


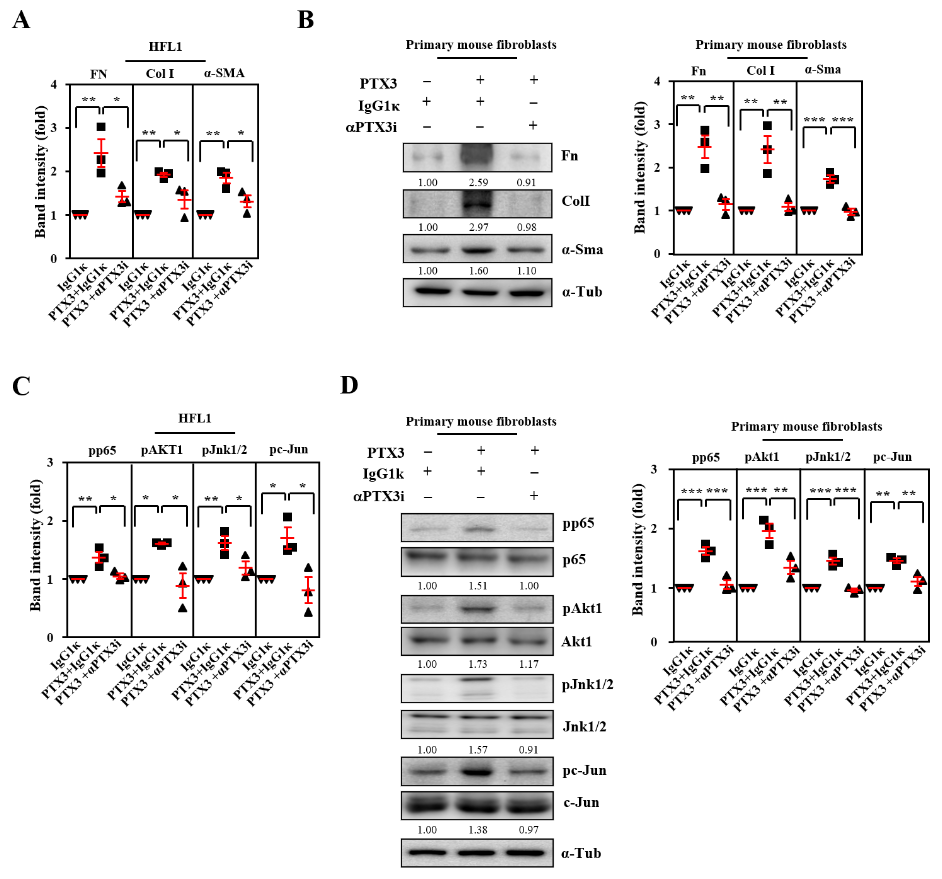


**(A)** HFL1 cells or **(B)** primary mouse lung fibroblasts were preincubated with IgG1κ and αPTX3i and then treated with PTX3. Quantitative analysis of the levels of Fibronectin, Collagen I and α-SMA in cells. **(C)** HFL1 cells or **(D)** primary mouse lung fibroblasts were incubated with IgG1κ and αPTX3i and then treated with PTX3 for 15 min. Quantitative analysis of the levels of pAKT1, pJNK, pc-Jun and pp65 in cells. Immunoblotting was replicated independently at least three times per experiment. All data are shown as the mean ± SEM. Differences among the groups were analyzed using unpaired two-tailed t tests. *p < 0.05, **p < 0.01, ***p < 0.001.

**Fig. S11. The response of PTX2 in PTX3-regulated pulmonary fibrosis.**


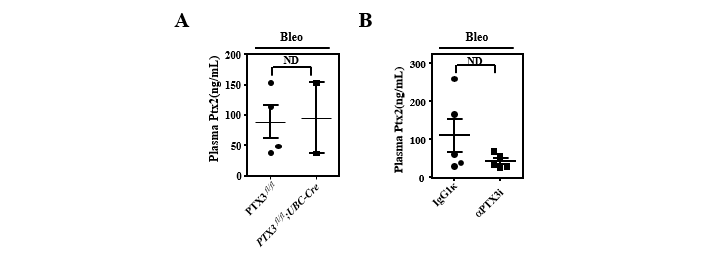


**(A)** Plasma Ptx2 concentrations were measured by ELISA in *Ptx3 ^fl/fl^* mice and *Ptx3 ^fl/fl^;UBC-Cre mice* following bleomycin administration. **(B)** Plasma Ptx2 concentrations were measured by ELISA in IgG1κ-treated mice and αPTX3i-treated mice following bleomycin administration. All data are shown as the mean ± SEM. Differences among the groups were analyzed using unpaired two-tailed t tests. ND: no difference.

**Fig. S12. Bleomycin-induced pulmonary fibrosis is attenuated in conditional Ptx3 pre-inactivated mice (A)** Scheme showing the experimental setup in tamoxifen‐induced conditional Ptx3‐knockout mice. Tamoxifen was firstly administered in both wild-type *(Ptx3 ^fl/fl^)* (n=7) and tamoxifen‐induced conditional Ptx3‐knockout *(Ptx3 ^fl/fl^;UBC-Cre)* (n=4) mice daily by intraperitoneal injection. After 5 days, *Ptx3 ^fl/fl^* mice and *Ptx3 ^fl/fl^;UBC-Cre* mice were intratracheally instilled with 2 mg/kg bleomycin (Bleo) on day 0 and were subsequently euthanized on day 28. **(B)** Lung tissue Ptx3 and plasma Ptx3 concentrations were measured in Ptx3 fl/fl mice and Ptx3 fl/fl;UBC-Cre mice on day 28 post bleomycin administration by ELISA. **(C)** Body weight was continually measured for 28 days after bleomycin administration. Day 0 weight was set as the relative standard. **(D)** Macroscopic view of the lungs at study endpoint. Representative hematoxylin and eosin (H&E), Sirius red and Masson’s trichrome staining in representative lung sections from *Ptx3 ^fl/fl^* mice and *Ptx3 ^fl/fl^;UBC-Cre* mice on day 28 after bleomycin administration. Scale bars are 200 µm. Quantitative analysis of alveolar area was performed on H&E-stained sections, and the area of fibrosis was quantified by Sirius red- and Masson’s trichrome-stained sections of lung tissue. **(E)** Lung hydroxyproline concentrations were measured using a hydroxyproline assay kit. Lung tissue was harvested from *Ptx3 ^fl/fl^* mice and *Ptx3 ^fl/fl^;UBC-Cre* mice after bleomycin administration. **(F)** Representative computed tomography slices of mouse lungs on day 28 post bleomycin administration. Quantitative analysis of lung injury was performed on micro-CT sections using CT-Analyzer software. All data are shown as the mean ± SEM. Differences among the groups were analyzed using unpaired two-tailed t tests. *p < 0.05, ***p < 0.001.


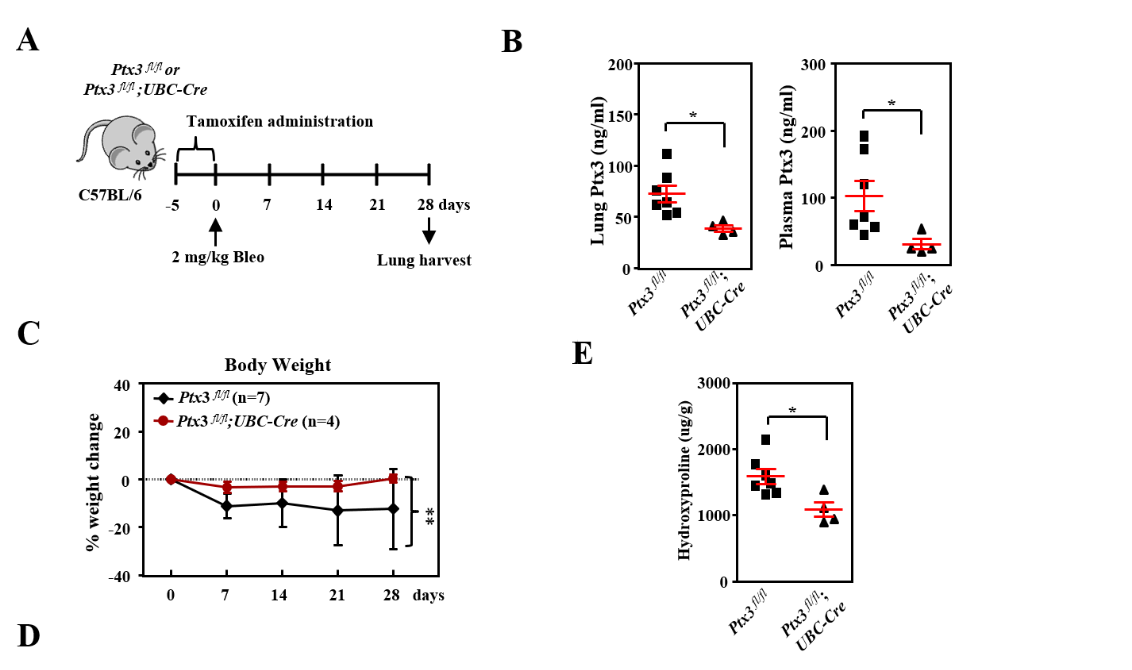


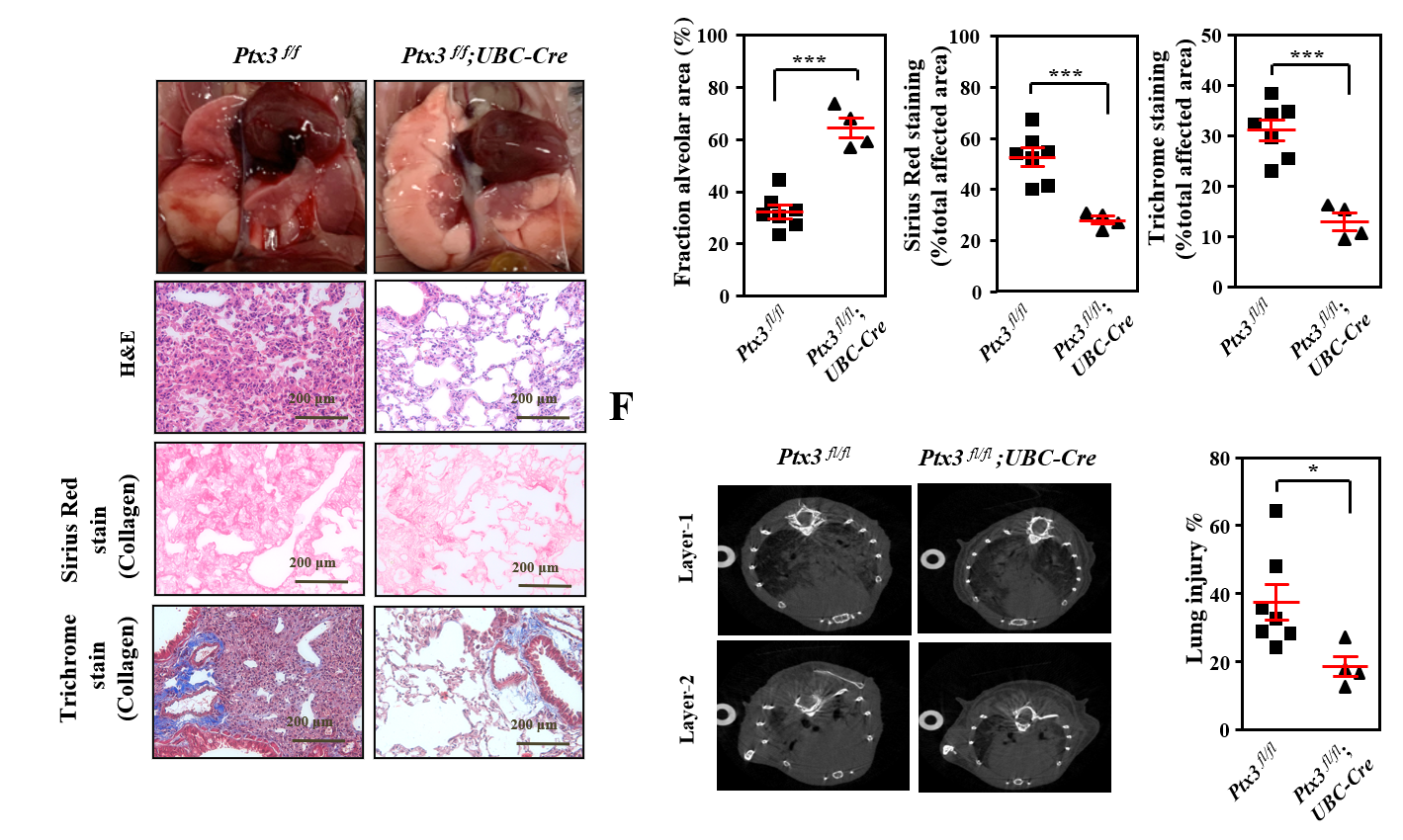


**Fig. S13. Preventative αPTX3i treatment significantly attenuates bleomycin-induced pulmonary fibrosis in mice.**


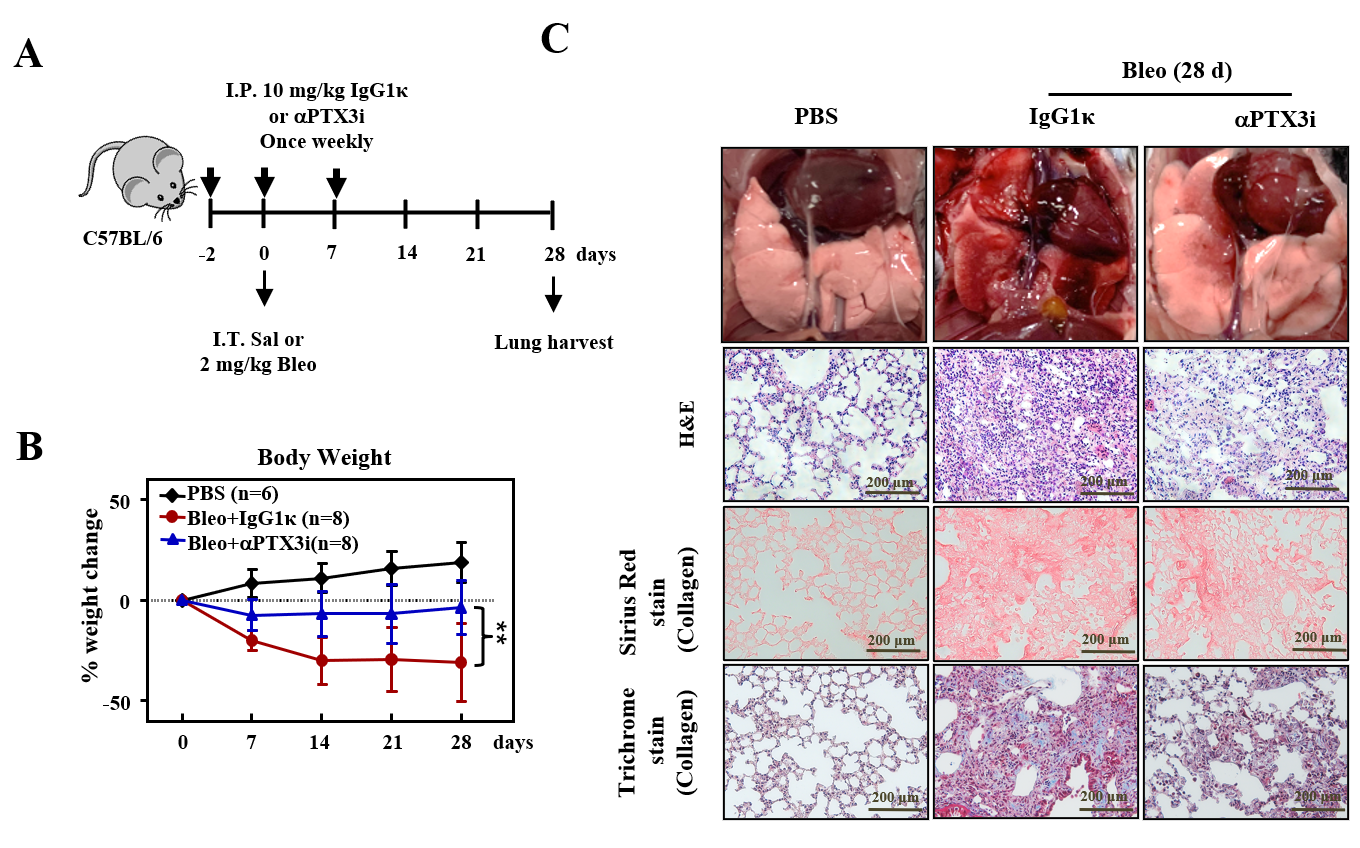


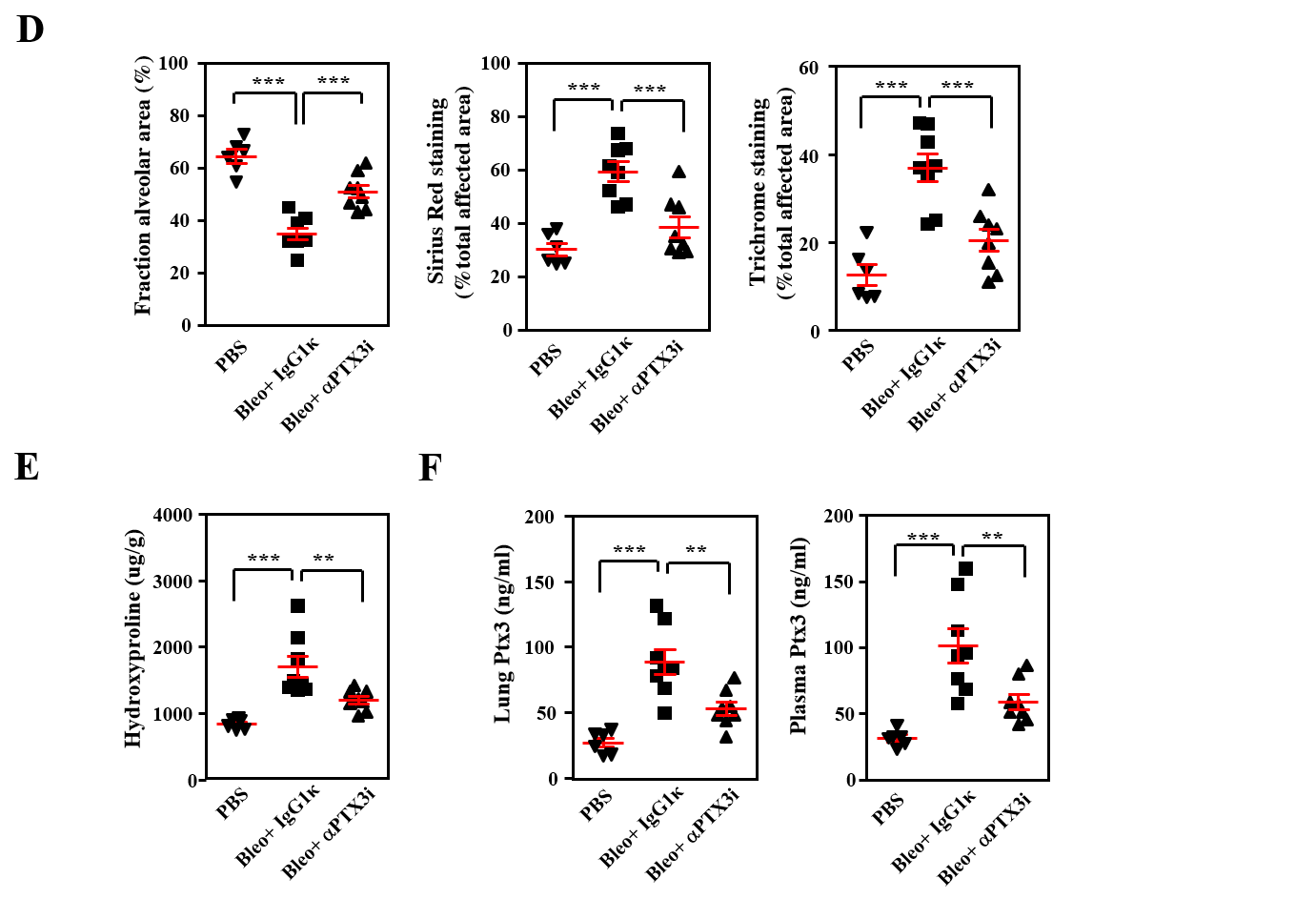


**(A)** Scheme showing the experimental flowchart. C57BL/6 mice were intratracheally instilled with PBS or 2 mg/kg bleomycin (Bleo) on day 0 and then euthanized on day 28. IgG1κ (10 mg/kg) or αPTX3i (10 mg/kg) was administered by intraperitoneal injection at two days ago. (PBS: n=6, Bleo+IgG1κ: n=8, Bleo+αPTX3i: n=8) **(B)** Body weight was continually measured for 28 days post bleomycin administration as a percentage of day 0 weight for each group as indicated. **(C)** Macroscopic view of the lungs at study endpoint. Representative hematoxylin and eosin (H&E), Sirius red and Masson’s trichrome staining in representative lung sections from IgG1κ-treated mice and αPTX3i-treated mice post bleomycin administration. Scale bars are 200 µm. **(D)** Quantitative analysis of the alveolar area was performed on H&E-stained sections. The area of fibrosis was quantified using Sirius red- and Masson’s trichrome-stained sections of lung tissue. **(E)** Lung hydroxyproline concentrations were measured using a hydroxyproline assay kit. Lung tissue was harvested from PBS-treated mice, Bleo+IgG1κ-treated mice and Bleo+αPTX3i-treated mice. **(F)** Lung tissue Ptx3 and plasma Ptx3 concentrations were measured in PBS-treated mice, Bleo+IgG1κ-treated mice and Bleo+αPTX3i-treated mice on day 28 post bleomycin administration by ELISA.

| Baseline characteristics | Patients without fILD  (N = 15) | Patients with fILD  (N =207) | *P*-value^1^ |
| --- | --- | --- | --- |
| Age, years | 63.8 (54.5 – 76.5) | 71.6 (64.1 – 79.7) | 0.040 |
| Body height, cm | 160.5 (152.0 – 168.0) | 160.0 (155.0 – 167.0) | 0.470 |
| Body weight, Kg | 61.0 (56.8 – 72.5) | 62.0 (55.0 – 70.0) | 0.540 |
| Body surface area^2^, m^2 | 1.65 (1.53 – 1.79) | 1.65 (1.54 – 1.78) | 0.896 |
| Body mass index, Kg/m^2 | 24.9 (23.2 – 27.9) | 23.8 (21.3 – 26.0) | 0.107 |
| Charlson comorbidity index | 4 (2 – 6) | 5 (3 – 6) | 0.219 |
| Plasma level of PTX3, ng/mL | 1.16 (1.03 – 1.54) | 1.36 (1.00 – 2.60) | 0.136 |
| Sex |  |  | 0.151 |
| Female | 7 (47) | 59 (29) |  |
| Male | 8 (53) | 148 (71) |  |
| Status of cigarette smoking |  |  | 0.649 |
| Yes | 2 (13) | 20 (10) |  |
| No | 13 (87) | 187 (90) |  |
| Types of fILD^3^ |  |  | - |
| IPF | - | 85 (41) |  |
| CPFE | - | 43 (21) |  |
| Idiopathic fibrosing NSIP | - | 15 (7) |  |
| CTD-related UIP | - | 39 (19) |  |
| CTD-related fibrosing NSIP | - | 23 (11) |  |
| CHP | - | 2 (1) |  |
| GAP stages of fILD |  |  | - |
| Stage 1 | - | 89 (43) |  |
| Stage 2 | - | 74 (36) |  |
| Stage 3 | - | 44 (21) |  |
| Values are presented in counts (% of group N) or median (inter-quartile range). Abbreviations: CHP, chronic hypersensitivity pneumonia; CPFE, combined pulmonary fibrosis and emphysema; CTD, connective tissue disease; fILD, fibrosing interstitial lung diseases; GAP, gender-age-physiology; IPF, idiopathic pulmonary fibrosis; NSIP, nonspecific interstitial pneumonia; PTX3; pentraxin 3; UIP, usual interstitial pneumonia. ^1^ *P*-values for the comparison between patients with and those without fILD. ^2^ The body surface area was derived using Du Bois method. ^3^ Subtypes of fILD were determined through multi-disciplinary discussions based on the radiographic features on high-resolution computed tomographic images and, when available, histologic features. | | | |

**Table S1. Baseline characteristics of the 222 enrolled subjects.**

**Table S2. Comparison of baseline plasma levels of pentraxin 3 among different subgroups as stratified according to age and disease severity.**

| Subgroups based on age strata | Patients without fILD | Patients with fILD | | | *P*-value^1^ |
| --- | --- | --- | --- | --- | --- |
|  |  | GAP stage 1 | GAP stage 2 | GAP stage 3 |  |
| All ages | 1.16 (1.03 – 1.54) | 1.25 (0.89 – 2.20) | 1.31 (1.05 – 2.53) | 1.97 (1.22 – 4.00) | 0.002 |
|  |  |  |  |  |  |
| Age < 65 | 1.05 (0.84 – 1.36) | 1.25 (0.89 – 2.56) | 1.33 (0.99 – 2.98) | 4.55 (1.17 – 11.08) | 0.204 |
| 65 ≤ Age | 1.54 (1.06 – 1.84) | 1.24 (0.88 – 1.94) | 1.28 (1.05 – 2.39) | 1.88 (1.22 – 3.88) | 0.026 |
|  |  |  |  |  |  |
| 40 ≤ Age < 60 | 1.08 (0.86 – 1.34) | 1.03 (0.87 – 2.07) | 2.46 (1.31 – 2.98) | 7.12 (single value) | 0.102 |
| 60 ≤ Age < 80 | 1.16 (1.04 – 1.69) | 1.33 (0.90 – 2.12) | 1.23 (1.03 – 2.14) | 1.80 (1.14 – 3.53) | 0.132 |
| 80 ≤ Age | - | 1.13 (0.83 – 3.41) | 1.49 (1.06 – 2.55) | 2.41 (1.27 – 5.55) | 0.263 |
|  |  |  |  |  |  |
| Values are plasma levels of pentraxin 3 in ng/mL, presented in medians (inter-quartile ranges). Abbreviations: fILD, fibrosing interstitial lung diseases; GAP, gender-age-physiology. ^1^ *P*-values for the comparison (using Kruskal-Wallis test) among the different subgroups for each age strata. | | | | | |
